# Identification and validation of clinical phenotypes in *Staphylococcus aureus* bloodstream infection and their association with mortality (FEN-AUREUS study)

**DOI:** 10.1016/j.eclinm.2025.103240

**Published:** 2025-05-07

**Authors:** Belén Gutiérrez-Gutiérrez, Belén Gallego-Mesa, Achim J. Kaasch, Matthias Riediger, Siegbert Rieg, Marta Trigo, Sonsoles Salto-Alejandre, Francisco Anguita-Santos, Ángela Cano, Andrea Prolo-Acosta, Salvador López-Cárdenas, María Teresa Pérez-Rodríguez, Francisco Javier Martínez-Marcos, Esperanza Merino-Lucas, Blanca Anaya-Baz, Ana Arizcorreta, Antonio Plata-Ciézar, Marco Piscaglia, Alexandra Aceituno, Lidia Romero-Calderón, Daniel Hornuss, Aurora Alemán-Rodríguez, Adelina Gimeno-Gascón, Esther Recacha, Julián Torre-Cisneros, Nicolás Merchante, Álvaro Pascual, Luis Eduardo López-Cortés, Jesús Rodríguez-Baño

**Affiliations:** a Unidad de Enfermedades Infecciosas y Microbiología, Hospital Universitario Virgen Macarena and Departamentos de Medicina y Microbiología, Universidad de Sevilla/Instituto de Biomedicina de Sevilla/CSIC, Seville, Spain; b CIBER de Enfermedades Infecciosas (CIBERINFEC). Instituto de Salud Carlos III, Madrid, Spain; c Institute of Medical Microbiology and Hospital Hygiene, Medical Faculty, Otto von Guericke University Magdeburg, Magdeburg, Germany; d Division of Infectious Diseases, Department of Medicine II, Medical Center, Faculty of Medicine, University of Freiburg, Freiburg, Germany; e Unidad de Enfermedades Infecciosas y Microbiología. Hospital Universitario de Valme, Instituto de Biomedicina de Sevilla (IBiS), Universidad de Sevilla, Sevilla, Spain; f Clinical Unit of Infectious Diseases, Microbiology and Parasitology/Instituto de Biomedicina (IBIS), Virgen del Rocío University Hospital/CSIC/University of Seville, Seville, Spain; g Servicio de Enfermedades Infecciosas, Hospital Universitario San Cecilio, Granada, Spain; h Unidad de Enfermedades Infecciosas, Hospital Universitario Reina Sofía, Universidad de Córdoba/Instituto Maimónides de Investigación Biomédica de Córdoba (IMIBIC), Córdoba, Spain; i Servicio de Enfermedades Infecciosas, Hospital Virgen de la Victoria, Málaga, Spain; j Unidad de Enfermedades Infecciosas y Microbiología Clínica, Hospital Universitario de Jerez/Departamento de Medicina y Cirugía, Universidad de Cádiz/Instituto de Investigación e Innovación Biomédica de Cádiz, Cádiz, Spain; k Servicio de Medicina Interna, Hospital Álvaro Cunqueiro, Vigo, Pontevedra, Spain; l Servicio de Enfermedades Infecciosas, Hospital Juan Ramón Jiménez, Huelva, Spain; m Infectious Diseases Unit, Dr. Balmis General University Hospital, Alicante Institute for Health and Biomedical Research (ISABIAL), Clinical Medicine Department, University Miguel Hernández of Elche, Alicante, Spain; n Unit of Infectious Diseases, Hospital Universitario Puerto Real/Institute of Biomedical Research and Innovation of Cádiz (INIBICA), Cádiz, Spain; o Unidad de Enfermedades Infecciosas del Hospital Universitario Puerta del Mar, Cádiz, Spain; p Servicio de Enfermedades Infecciosas, Hospital Regional de Málaga, Spain; q Department of Infectious Diseases, ASST FBF Sacco, Milán, Italy; r Hospital Universitario Torrecárdenas, Almería, Spain

**Keywords:** *Staphylococcus aureus*, Phenotypes, Mortality, Bacteraemia, Clinical profiles

## Abstract

**Background:**

*Staphylococcus aureus* bacteraemia (SAB) is heterogeneous in patients and infection-related features. The aim of the study was to identify clinical phenotypes among patients with SAB, to evaluate their association with mortality, and to derive and validate a simplified probabilistic model for phenotypes assignment.

**Methods:**

Phenotypes were derived using two-stage cluster analysis of 2128 patients from the ISAC cohort (recruited between 2013 and 2015), analysing 62 variables. Cox regression assessed phenotype–mortality associations. Logistic regression was employed to develop a simplified probabilistic model for sub-phenotype allocation, validated in two external international cohorts: INSTINCT (1217 patients, recruited between 2006 and 2011) and FEN-AUREUS (1185 patients, recruited between January 2021 and October 2024). The association between sub-phenotypes and 30-day mortality in the validation cohorts was also assessed.

**Findings:**

Cluster analysis identified three clinical phenotypes based on the probable portal of entry: A (skin and soft tissues), 458 cases; B (vascular device-associated), 573 cases; and C (other portals of entry or unknown), 1097 cases. Their 30-day mortality was significantly different (13·1%, 18·2% and 25·3%, respectively, p < 0·001). Each phenotype contained two sub-phenotypes with differing characteristics and mortality risks. Also, three phenotypes were found in the INSTINCT cohort, which clustered on the same portals of entry, with two sub-phenotypes in each. When the simplified probabilistic model was applied, the sub-phenotypes showed significant associations with 30-day mortality in both validation cohorts. In INSTINCT, the aHRs were 1·93 (A2 vs A1), 3·40 (B2 vs B1), and 3·04 (C2 vs C1). In FEN-AUREUS, the aHRs were 2·02 (A2 vs A1), 2·11 (B2 vs B1), and 2·44 (C2 vs C1).

**Interpretation:**

Patients with SAB can be classified into phenotypes and sub-phenotypes, each exhibiting considerable variations in mortality rates. To facilitate clinical application, a validated open-access algorithm and calculator for phenotype and sub-phenotype assignment have been developed, enabling their use at the time of SAB confirmation. This tool aims to support timely and personalised patient care.

**Funding:**

10.13039/501100004587Instituto de Salud Carlos III, Spanish Ministry of Science, Innovation and Universities (PI21/01801).

**Research in context**

**Evidence before this study**

We searched PubMed, Scopus, and medRxiv up to October 2024 using the terms [“*Staphylococcus aureus*” OR “*S. aureus*”] AND [“phenotypes” OR “clinical features”] with no language restrictions to identify any study characterising clinical phenotypes in bloodstream infections caused by *Staphylococcus aureus* (SAB). To our knowledge, only one prior study, based on the analysis of 1430 cases and published online in June 2024, attempted to classify SAB patients into phenotypes. No further studies explicitly characterising SAB phenotypes or investigating their association with outcomes were identified.

**Added value of this study**

Based on a cohort of 2128 patients, we identified, through comprehensive cluster analysis of clinical and laboratory data collected within the first 24 h from blood culture extraction, three distinct clinical phenotypes in SAB patients. Additionally, two sub-phenotypes were identified within each phenotype. Both the phenotypes and sub-phenotypes obtained differed in acquisition type, comorbidities, bacteraemia source, laboratory data, and antibiotic susceptibility, and showed significantly different 30-day mortality risks. Based on these results, we developed an algorithm using simplified probabilistic models that easily assign patients to phenotypes/sub-phenotypes, which was validated in two external cohorts of 1217 and 1185 cases, respectively. This study provides a novel framework to stratify SAB patients into phenotypes and sub-phenotypes with different mortality risks, enabling clinicians worldwide to identify high-risk patients requiring closer monitoring. To this end, we have made these models publicly and freely available through an online tool at http://fen-aureus.com.

**Implications of all the available evidence**

The identification and validation of phenotypes and sub-phenotypes in SAB represents a step forward in understanding this heterogeneous type of infection. These phenotypes and sub-phenotypes could be used, in addition to helping clinicians identify high-risk patients, to explore differences in the underlying pathophysiological mechanisms of phenotypes and sub-phenotypes, refine patient selection for clinical trials, or optimise therapeutic strategies.

## Introduction

*Staphylococcus aureus* is one of the most frequent microorganisms causing disease in humans and a leading cause of bloodstream infections globally.^1^^,^^2^ *S. aureus* bacteraemia (SAB) results in substantial morbidity and healthcare costs, with complications being common and mortality rates ranging between 20% and 40%.^1^, ^2^, ^3^

Bacteraemic infections due to *S. aureus* are typically very heterogeneous in terms of patients’ underlying conditions, pathogen characteristics, portals of entry, type of acquisition, site of infection and acute severity.^1^^,^^4^^,^^5^ Moreover, there may be intrinsic clinical heterogeneity in SAB that extends beyond these variables,^6^ making the establishment of management standards of care very complex.^7^^,^^8^ This is reflected in the large practice variations across the world.^9^ It also poses specific difficulties for both designing and interpreting the results of randomised trials.^6^^,^^10^

We hypothesise that patients who develop SAB can be classified into clinical phenotypes associated with prognosis. The phenotypes may be the consequence of subjacent subtle different pathophysiological mechanisms and may also be associated with outcomes, which therefore may have important clinical and management implications. As examples in infectious diseases, clinical phenotypes have been identified in patients with sepsis^11^ or COVID-19.^12^

The objective of this study was to identify SAB phenotypes, investigate their association with mortality, and develop simplified probabilistic models for assigning patients to these phenotypes, with the aim of facilitating their application in clinical practice.

## Methods

### Study design and population

**Fig. 1:**
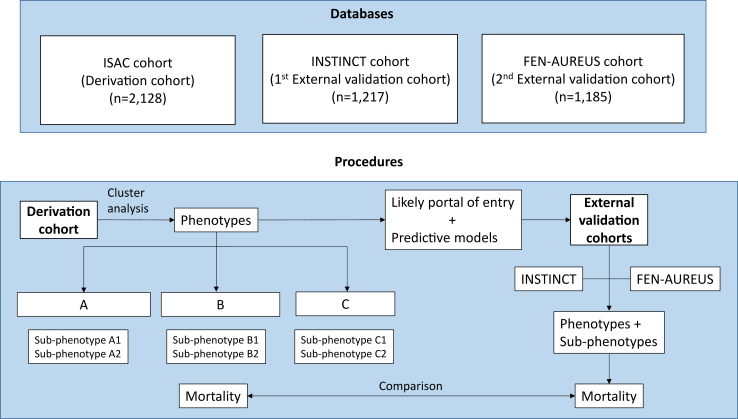
**Databases used and procedures performed.**

This study is part of the FEN-AUREUS project (clinicaltrials.gov identifier: NCT06574399). Clinical phenotypes in patients with SAB were derived using data from the International *Staphylococcus aureus* Collaboration (ISAC) prospective cohort (2128 patients recruited from 2013 to 2015 from 11 hospitals in 5 countries); the correlation of phenotypes with 30-day mortality was assessed, and a simplified probabilistic model for phenotype allocation was derived. The phenotypes allocation model and the association of phenotypes with mortality were externally validated in the INvasive *S. aureus* INfections CohorT (INSTINCT), a prospective cohort involving 1217 patients from 2 hospitals, recruited from 2006 to 2011. The methodological aspects of both cohorts were previously published.^13^^,^^14^ Finally, the FEN-AUREUS prospective cohort, performed in 19 centres from two countries from January 2021 to October 2024 and including 1185 patients, was also used for contemporary external validation. In all cohorts, patients were eligible for the study if they were at least 18 years of age and had at least one blood culture positive for *S. aureus* with accompanying clinical symptoms and signs of infection. Written informed consent was obtained from all participants prior to inclusion in each of the three prospective cohorts (ISAC, INSTINCT, and FEN-AUREUS). An overview of the analyses performed in the derivation and validation cohorts is presented in Fig. 1.

The FEN-AUREUS project was approved by the Ethics Committee of Biomedical Research of Andalusia (1428-N-22). The study is reported according to the STROBE recommendations.

### Variables and definitions

The variables considered to derive the clinical phenotypes were collected within the first 24 h from blood culture extraction and included age, gender, comorbidities, blood chemistry and blood cell counts, likely *S. aureus*-portal of entry, source of bacteraemia, severity of infection and methicillin-susceptibility. The full list of variables is shown in Table 1. Additionally, variables related to early clinical management, such as antibiotic treatment, source control or ID consultation, and centre, were included. The main endpoint was 30-day all-cause mortality; patients discharged before day 30 were contacted by phone at the end of the follow-up period to assess their status.

**Table 1:** Features and outcomes of patients in the derivation cohort and in the phenotypes obtained.

Factor	A^a^ (n = 458)	B^b^ (n = 573)	C^c^ (n = 1097)	p-value
**Demographics**				
Female sex	155 (33·8)	212 (37·0)	410 (37·4)	0·44
Median age in years (IQR)	66 (53–77)	62 (50–73)	67 (54–78)	<0·001
**Comorbidities**				
Myocardial infarction	77 (16·8)	96 (16·8)	147 (13·4)	0·09
Peripheral vascular disease	73 (15·9)	55 (9·6)	89 (8·1)	<0·001
Dementia	43 (9·4)	19 (3·3)	84 (7·7)	<0·001
Chronic lung disease	54 (11·8)	91 (15·9)	150 (13·7)	0·14
Leukaemia	13 (2·8)	22 (3·8)	26 (2·4)	0·23
Lymphoma	13 (2·8)	35 (6·1)	31 (2·8)	0·002
Solid cancer without metastasis	36 (7·9)	54 (9·4)	99 (9·0)	0·68
Metastatic solid cancer	16 (3·5)	71 (12·4)	96 (8·8)	<0·001
Chronic renal disease	100 (21·8)	201 (35·1)	228 (20·8)	<0·001
Cerebrovascular disease				
No cerebrovascular disease	387 (84·5)	492 (85·9)	942 (85·9)	0·91
Mild or no residual neurological defect	49 (10·7)	58 (10·1)	105 (9·6)	
Hemiplegia	22 (4·8)	23 (4·0)	50 (4·6)	
Chronic liver disease				
No liver disease	397 (86·7)	516 (90·1)	916 (83·5)	0·003
Mild	33 (7·2)	29 (5·1)	80 (7·3)	
Moderate or severe	28 (6·1)	28 (4·9)	101 (9·2)	
Diabetes mellitus				
Not diabetic	278 (60·7)	397 (69·3)	800 (72·9)	<0·001
Without end-organ damage	95 (20·7)	92 (16·1)	191 (17·4)	
With end-organ damage	85 (18·6)	84 (14·7)	106 (9·7)	
HIV infection	9 (2·0)	6 (1·0)	15 (1·4)	0·45
**Invasive procedures or treatment**				
Hemodialysis	37 (8·1)	134 (23·4)	67 (6·1)	<0·001
Systemic corticosteroid for more than 2 weeks	26 (5·7)	54 (9·4)	63 (5·7)	0·011
Neutropenia	2 (0·4)	25 (4·4)	35 (3·2)	0·001
Current chemotherapy	5 (1·1)	84 (14·7)	78 (7·1)	<0·001
Immunesuppressive therapy	27 (5·9)	38 (6·6)	58 (5·3)	0·53
Organ or bone marrow transplantation	14 (3·1)	24 (4·2)	43 (3·9)	0·62
Previous surgery (last month)	4 (0·9)	1 (0·2)	3 (0·3)	0·14
Vascular catheter at onset	77 (16·8)	457 (79·8)	298 (27·2)	<0·001
**Acquisition of infection**				
Community-acquired	181 (39·5)	30 (5·2)	471 (42·9)	<0·001
Healthcare-associated	152 (33·2)	200 (34·9)	274 (25·0)	<0·001
Nosocomial	126 (27·5)	343 (59·9)	352 (32·1)	<0·001
**Main site of infection**^d^				
Skin and soft tissues excluding surgical wound and deep tissue	192 (41·9)	17 (3·0)	39 (3·6)	<0·001
Surgical wound	88 (19·2)	7 (1·2)	0 (0·0)	<0·001
Central venous catheter (including PICC)	7 (1·5)	252 (44·0)	0 (0·0)	<0·001
Peripheral venous catheter	11 (2·4)	182 (31·8)	0 (0·0)	<0·001
Infected intravascular thrombus	10 (2·2)	14 (2·4)	11 (1·0)	0·05
Implanted vascular device	3 (0·7)	95 (16·6)	44 (4·0)	<0·001
Native heart valve	21 (4·6)	19 (3·3)	82 (7·5)	0·003
Prosthetic heart valve	9 (2·0)	4 (0·7)	27 (2·5)	0·04
Epidural or intraspinal empyema	5 (1·1)	4 (0·7)	37 (3·4)	<0·001
Vertebral bone/disc	18 (3·9)	7 (1·2)	87 (7·9)	<0·001
Native joint	33 (7·2)	11 (1·9)	76 (6·9)	<0·001
Prosthetic joint	13 (2·9)	3 (0·5)	28 (2·6)	0·01
Other bone-related source	42 (9·2)	1 (0·2)	20 (1·8)	<0·001
Deep tissue infection or abscess	49 (10·7)	6 (1·0)	100 (9·1)	<0·001
Pneumonia	28 (6·1)	9 (1·6)	180 (16·4)	<0·001
Source not established	25 (5·5)	17 (3·0)	362 (33·0)	<0·001
Other sources	20 (4·4)	27 (4·7)	44 (4·0)	0·73
**Other infection features**				
Previous non-bacteraemic *S. aureus* infection in the last 12 weeks	18 (3·9)	21 (3·7)	58 (5·3)	0·25
More than 2 days with symptoms	144 (31·4)	74 (12·9)	255 (23·2)	<0·001
Median days from admission until confirmation^e^ of *S. aureus* bacteremia (IQR)	0 (0–5)	5 (0–13)	1 (0–6)	<0·001
**Antibiotic susceptibility**				
Methicillin-resistant	105 (22·9)	94 (16·4)	175 (16·0)	0·003
**Physical examination data**				
Hypotension (arterial blood pressure<70 mmHG)	96 (21·1)	111 (19·4)	236 (21·5)	0·59
Inotropes use	179 (16·3)	59 (12·9)	56 (9·8)	0·001
Lowest Glasgow score, median (IQR)	15 (14–15)	15 (14–15)	15 (13–15)	<0·001
Highest temperature (°C), median (IQR)	38·3 (37·4–38·8)	38·5 (38·1–39·0)	38·2 (37·5–38·7)	<0·001
Maximum heart rate (bpm), median (IQR)	98 (89–111)	98 (91–113)	98 (90–115)	0·6
**Laboratory analysis**				
Lowest O2 saturation in %, median (IQR)	94 (93–96)	95 (93–96)	94 (92–96)	0·001
Highest FiO2%, median (IQR)	24 (21–40)	26 (21–35)	28 (21–40)	0·02
Serum bilirubin (mg/dL), median (IQR)	0·58 (0·35–0·90)	0·75 (0·47–1·12)	0·70 (0·44–1·08)	0·53
Serum creatinine (mg/dL), median (IQR)	1·24 (0·85–1·90)	1·19 (0·80–3·50)	1·16 (0·79–2·00)	0·019
Serum CRP (mg/L), median (IQR)	156·0 (54·5–231·3)	103·0 (36·0–163·0)	118·0 (37·0–191·0)	<0·001
Blood white cell count (×10^3^/μL), median (IQR)	13·0 (9·0–16·9)	10·9 (7·3–14·5)	12·8 (8·6–16·8)	<0·001
Neutrophil count (×10^3^/μL), median (IQR)	10·8 (7·5–14·8)	9·3 (6·0–12·6)	10·7 (7·1–14·5)	<0·001
Lymphocyte count (×10^3^/μL), median (IQR)	1·2 (0·7–1·7)	0·85 (0·45–1·25)	0·95 (0·55–1·35)	<0·001
Platelet count (×10^3^/μL), median (IQR)	220 (142–298)	185 (123–252)	188 (114–256)	<0·001

Data are expressed as No. (%) except where specified.

a Portals of entry: skin or soft tissue infection unrelated to surgical wounds: 324 cases (70·7%); surgical wounds: 134 cases (29·3%).

b Portals of entry: central vascular catheter (including PICC): 271 cases (47·3%); peripheral vascular catheter (including arterial line): 206 cases (35·9%); other implanted vascular device (e.g., pacemaker, stent, graft) with early-onset infections following device implantation: 96 cases (16·8%).

c Portals of entry: respiratory tract: 198 cases (18·8%); Genito-urinary tract: 123 cases (11·2%); Portal not known: 776 cases (70·7%).

d More than one allowed.

e Confirmation refers to the moment when blood culture results become available.

The probable portal of entry for SAB refers to the anatomical site through which the pathogen is believed to have entered the bloodstream. This was determined based on specific criteria (Supplementary Table S1), with only permitted one portal per case. They were classified as vascular catheter-related, skin and soft tissues, respiratory tract, and genito-urinary tract. If no probable portal could be determined, the entry was classified as unknown.

The site of infection refers to the primary organ site where the infection was located at the time the first positive blood culture was taken. The site of infection might or might not coincide with the portal of entry (e.g., a vascular catheter may be the portal of entry in a patient who later develops knee arthritis as the site of infection). Eventually, more than one source could be identified in a patient. Sources were determined based on clinical data, imaging studies, and isolation of the microorganism in specific source-related samples. Sources considered are listed in Supplementary Table S1.

Source control was defined as follows: if the bacteraemia was associated with a removable intravascular device, the source was considered controlled if the device was removed within 3 days. Alternatively, if the bacteraemia was related to a removable non-device source, such as an abscess, the source was considered controlled if the non-device related source was addressed (e.g., drained or surgically removed) within the same timeframe.

### Missing data and phenotypes derivation

The proportion of missing data per variable in the ISAC cohort is shown in the appendix (Supplementary Table S2); after discarding the hypothesis that data was missing completely at random by Little's MCAR test, missing data were completed by multiple imputation using the Markov chain Monte Carlo method.

To identify the phenotypes in the derivation cohort (ISAC cohort), we first assessed the distribution of values and correlations among variables using the chi-square test for categorical variables and Pearson's correlation coefficient for continuous variables, after verifying assumptions of normality and linearity. When these assumptions were not fulfilled, a Spearman's rank correlation matrix was used as an alternative to evaluate associations and detect potential collinearity between continuous variables. When two variables were highly correlated, one of them was excluded to eliminate collinearity A total of 62 variables were included to derive the clinical phenotypes. As the aim was to explore the potential existence of phenotypes, no preselection of variables was performed. An unsupervised two-step cluster analysis was then conducted using both continuous and categorical variables, allowing the optimal number of clusters to emerge naturally without imposing any predefined assumptions. This approach included a pre-clustering step based on a log-likelihood distance measure, followed by hierarchical clustering. The optimal number of clusters was automatically determined using Schwarz's Bayesian Information Criterion (BIC). To assess the quality and robustness of the clusters, silhouette analysis was applied. A sensitivity analysis excluding variables with more than 30% missing data was performed to validate the reliability of the identified phenotypes. The characteristics of patients across the identified phenotypes were compared using the Chi-square test for categorical variables and either ANOVA or the Kruskal–Wallis test for continuous variables, depending on whether the assumption of normality was met. Because the phenotypes were defined by the portal of entry, we then performed a new two-step cluster analysis for each of the phenotypes obtained, using the same methodology described above, to identify sub-phenotypes within each phenotype. The characteristics of patients in each of these sub-phenotypes were also analysed.

### Derivation and external validation of a parsimonious predictive models for phenotypes

Although assignment to phenotypes was straightforward, the high number of variables considered and the complexity in their distribution in the different sub-phenotypes make assigning patients to them inapplicable in clinical practice. To solve this, we developed probabilistic predictive models for sub-phenotypes assignment by performing one logistic regression analysis in the ISAC cohort per phenotype, with sub-phenotype as the outcome variable. Variables included in the multivariable models were those with a p-value <0·20 in the corresponding univariable logistic regression analyses. We used the variance inflation factor value to detect potential collinearity, and interactions were tested. The variables were selected using a manual backward selection process. The predictive ability of the final models for observed phenotypes assignment was checked by calculating their area under the receiver operating characteristic curves (AUROC) with 95% confidence intervals (CI).

These sub-phenotype-assignment predictive models were applied to patients in the INSTINCT and FEN-AUREUS cohort for their external validation; because the model-derived formula used for the assignment probabilities calculation corresponds with a binary logistic regression, each patient within a phenotype was assigned to sub-phenotype 1 or 2 according to whichever had a higher probability (>50%). We then assessed the distribution of clinical variables across the assigned phenotypes and sub-phenotypes.

### Prognostic assessment of the phenotypes and sub-phenotypes

Mortality until day 30 in patients belonging to the different phenotypes and sub-phenotypes in the derivation cohort (ISAC) was plotted using Kaplan–Meier curves and compared by the log-rank test. The association between sub-phenotypes and 30-day mortality was adjusted using Cox regression models that included management variables and centre only if they showed an association with 30-day mortality (p < 0·20) in the univariable analyses. These analyses were repeated in the external validation cohorts INSTINCT and FEN-AUREUS for the assigned phenotypes and sub-phenotypes. All analyses were performed with IBM SPSS Statistics 29, SPM 8·3 and R software version 4.4.1.

### Role of the funding source

The funders of the study had no role in study design, data collection, data analysis, data interpretation, or writing of the report. All authors had access to all the data. The corresponding author had final responsibility for the decision to submit for publication.

## Results

### Derivation of phenotypes

The features of the 2128 patients in the ISAC cohort, used for derivation of phenotypes, was previously reported in detail.^13^ The cluster analysis identified 3 clinical phenotypes based on the portal of entry: phenotype A (portal of entry: skin and soft tissues), 458 cases (21·5%); phenotype B (portal of entry: vascular catheter), 573 cases (26·9%); and phenotype C (others or unknown portal of entry), 1097 cases (51·6%). The silhouette score was 0·61, indicating good quality of clustering. The features of the patients, according to the phenotypes, are shown in Table 1. Overall, compared to patients in the other two phenotypes, those in phenotype A had fewer days of hospitalisation before developing bacteraemia, higher platelet counts, serum creatinine levels, CRP values and neutrophils count by the time of blood culture extraction, and a higher proportion of methicillin-resistant isolates. In contrast, patients in phenotype B were younger and exhibited a higher frequency of cancer, neutropenia, chronic renal disease, and the presence of a vascular catheter at the onset of infection, with SAB more frequently being nosocomial. The duration of symptoms prior to blood culture was shorter than in the other phenotypes. They also had a longer history of prior hospitalisation. Patients classified in phenotype C showed a higher percentage of community-acquired SAB, liver disease, and pneumonia as a source, and lower oxygen saturation.

In each of these phenotypes, the two-step cluster analysis was repeated, which allowed identifying two sub-phenotypes with each of them: sub-phenotypes A1 and A2; B1 and B2; C1 and C2. Sub-phenotypes differed in acquisition type, comorbidities, bacteraemia source, laboratory data, and methicillin-resistance (Supplementary Tables S3–S5). In phenotype A, sub-phenotype A1 showed a moderately severe profile, with fewer comorbidities but higher CRP levels. In contrast, A2 included higher rates of renal disease, diabetes mellitus with end-organ damage, methicillin-resistance, the highest platelet and creatinine levels, and the lowest lymphocyte count. Within phenotype B, sub-phenotype B1 exhibited moderate complexity, characterized by a lower prevalence of nosocomial infections, the highest oxygen saturation, and lowest white blood cell and neutrophil counts. Meanwhile, B2 was more severe, with a high rate of nosocomial infection, infections associated with central venous catheters, and higher methicillin resistance. Finally, among patients in phenotype C, sub-phenotype C1 was characterised as mild, with few chronic conditions, whereas C2 included older patients with multiple age-related comorbidities and the highest percentage of unknown foci (Supplementary Figure S1).

### 30-Day mortality in phenotypes and sub-phenotypes of the derivation cohort (ISAC)

**Fig. 2:**
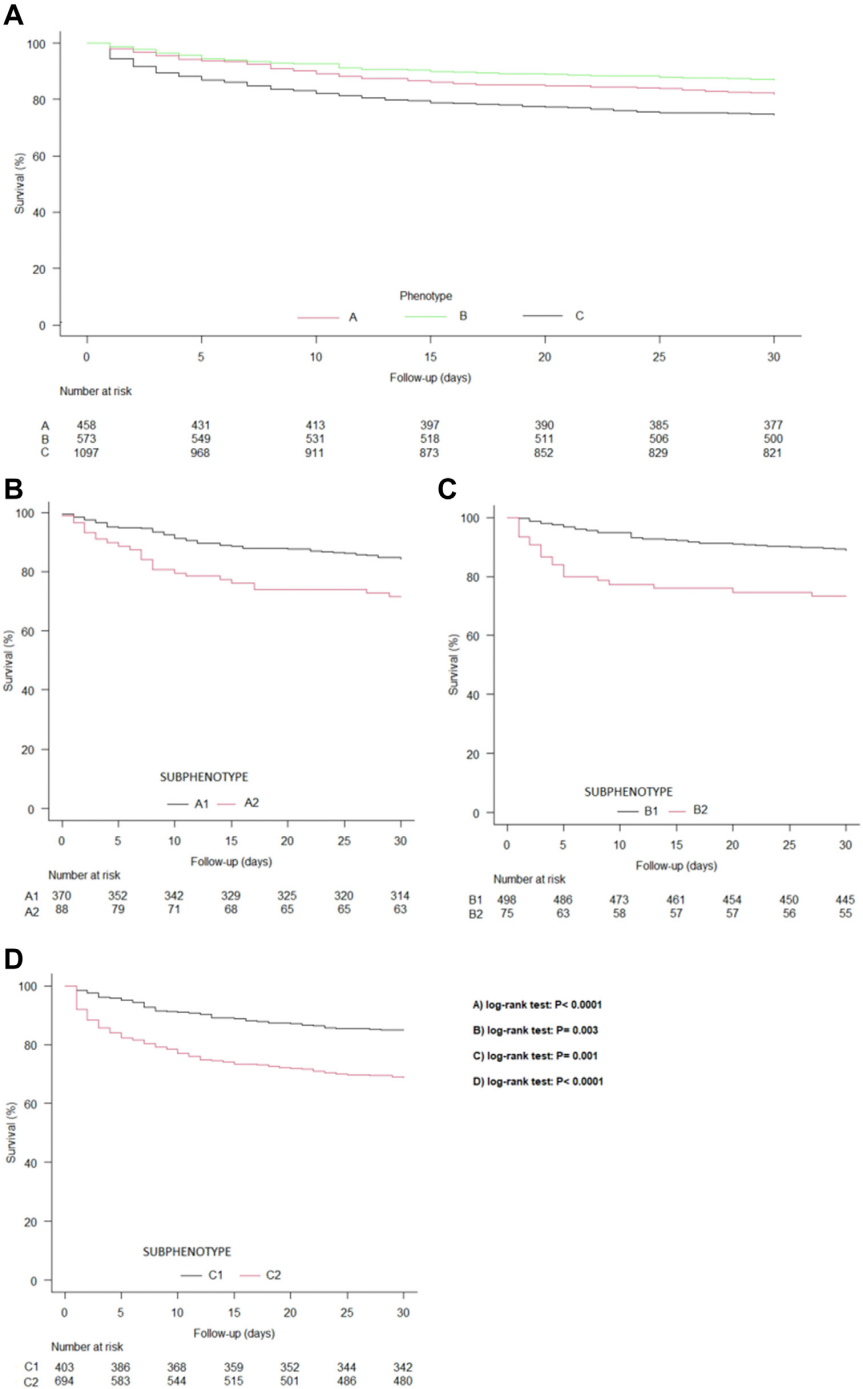
**Kaplan–Meier survival analysis: 30-day mortality by phenotype and sub-phenotype in the derivation cohort.** A) Kaplan–Meier survival analysis: 30-day mortality by phenotype based on the likely portal of entry in the derivation cohort. B) Kaplan–Meier Survival Analysis: 30-Day mortality by sub-phenotype in patients belonging to the A phenotype in the derivation cohort. C) Kaplan–Meier Survival Analysis: 30-Day mortality by sub-phenotype in patients belonging to the B phenotype in the derivation cohort. D) Kaplan–Meier Survival Analysis: 30-Day mortality by sub-phenotype in patients belonging to the C phenotype in the derivation cohort.

The three phenotypes were associated with different 30-day mortality rates: 18·1% (83/458), 13·1% (75/573) and 25·3% (277/1097) for phenotypes A, B and C, respectively (log-rank test p < 0·001) (Fig. 2A, Table 2). The sub-phenotypes also exhibited significant differences in 30-day mortality within each phenotype: A1, 15·7% (58/370) *vs* A2, 28·4% (25/88), p = 0·003; B1, 11·0% (55/498) *vs* B2, 26·7% (20/75), p = 0·001; and C1, 15·1% (61/403) *vs* C2, 31·1 (216/694), p < 0·001 (Fig. 2B, C, and D, Table 2).

**Table 2:** 30-Day mortality rates by phenotypes and sub-phenotypes in the ISAC derivation cohort and in the INSTINCT and FEN-AUREUS external validation cohorts.

Phenotype	Mortality (n/N; %)	Sub-phenotype	Mortality (n/N; %)	p (log-rank test)
**ISAC cohort**				
A	83/458 (18·1)	A1	58/370 (15·7)	0·003
		A2	25/88 (28·4)	
B	75/573 (13·1)	B1	55/498 (11·0)	0·001
		B2	20/75 (26·7)	
C	277/1097 (25·3)	C1	61/403 (15·1)	<0·0001
		C2	216/694 (31·1)	
**INSTINCT cohort**				
A	37/243 (15·2)	A1	20/166 (12·0)	0·04
		A2	17/77 (22·1)	
B	61/458 (13·3)	B1	38/383 (9·9)	<0·0001
		B2	23/75 (30·7)	
C	139/516 (26·9)	C1	21/165 (12·7)	<0·0001
		C2	118/351 (33·6)	
**FEN-AUREUS cohort**				
A	69/283 (24·4)	A1	31/181 (17·1)	0·0002
		A2	38/102 (37·3)	
B	37/573 (16·9)	B1	76/505 (15·0)	0·0007
		B2	21/68 (30·9)	
C	94/329 (28·6)	C1	19/123 (15·4)	<0·0001
		C2	75/206 (36·4)	

The association of A2, B2 and C2 with higher 30-day mortality was confirmed in multivariable Cox regression analyses conducted after adjusting for clinical management variables such as source control, infectious diseases consultation and antimicrobial treatment, as well as for centre effect. The adjusted hazard ratios (aHR) for mortality were 1·86 (95% CI 1·16–2·99, p = 0·01) for A2 respect to A1; 2·50 (95% CI 1·47–4·26, p = 0·0007) for B2 respect to B1; and 1·97 (95% CI 1·47–2·62, p < 0·0001) for C2 respect to C1 (Table 3). Additionally, consultation by an infectious diseases specialist and source control within day 3 were also protective for mortality across all three phenotypes.

**Table 3:** Multivariable cox regression of 30-day mortality for each of the identified phenotypes in the derivation cohort.

Variable	A phenotype		B phenotype		C phenotype	
	aHR (95% CI)	p	aHR (95% CI)	p	aHR (95% CI)	p
ID consultation	0·51 (0·31–0·84)	0·01	0·50 (0·30–0·84)	0·009	0·35 (0·27–0·44)	<0·0001
Source control within 3 days	0·39 (0·18–0·84)	0·02	0·45 (0·28–0·71)	0·001	0·34 (0·20–0·58)	<0·0001
Sub-phenotype 2^a^	1·86 (1·16–2·99)	0·01	2·50 (1·47–4·26)	0·0007	1·97 (1·47–2·62)	<0·0001
High risk centre^b^			1·90 (1·10–3·28)	0·02	1·34 (1·00–1·80)	0·05

a A2 sub-phenotype with respect to A1 in A phenotype; B2 sub-phenotype with respect to B1 in B phenotype; and C2 sub-phenotype with respect to C1 in C phenotype.

b In Phenotype A, “high-risk centre” was not significantly associated with mortality in the final model.

### Predictive models for sub-phenotypes assignment

To facilitate the clinical application of phenotype and sub-phenotype identification, we performed a multivariable logistic regression analysis within each phenotype using the sub-phenotype as dependent variable. The final multivariable logistic regression models are detailed in Table 3 and included 7 variables for classifying patients in sub-phenotypes A1 or A2, 5 for B1 or B2, and 14 for C1 or C2. These models exhibited very good predictive ability, with AUROCs (95% CI) of 0·86 (0·82–0·91), 0·88 (0·83–0·92), and 0·89 (0·86–0·91), respectively (Supplementary Table S6).

An online, freely available application to assign SAB patients to sub-phenotypes based on the developed probabilistic models is accessible at https://fen-aureus.com/.

### Validation in external cohorts

We replicated the two-phase cluster analysis in the INSTINCT validation cohort, confirming the presence of three phenotypes, each subdivided into two sub-phenotypes. The phenotypes also clustered based mostly on the portal of entry: the first included 355 cases, with 80·5% having skin and soft tissues as the portal of entry; the second comprised 432 cases, 99·8% of which had a vascular catheter as the portal of entry; and the third phenotype included 430 cases, with 95·1% associated with an unknown or other portal of entry (Supplementary Table S7).

**Fig. 3:**
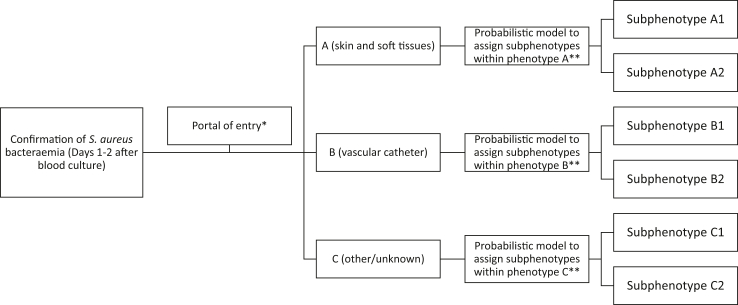
**Proposed clinical management algorithm fortheclassification of patients with *S. aureus* bacteraemia based on identifiedphenotypes and sub-phenotypes**. ∗See definitions to consider a probable portal of entry in Supplementary material. ∗∗Visit webpage: https://fen-aureus.com/ for phenotypes and subphenotypes assignment.

Phenotypes, sub-phenotypes, and the corresponding probabilistic models were exclusively derived from the derivation cohort (ISAC) and then directly applied to the external validation cohorts INSTINCT and FEN-AUREUS. For phenotype assignment, patients were classified based on their portal of entry. Subsequently, sub-phenotypes were assigned according to the proposed algorithm (Fig. 3). The resulting distribution in the INSTINCT cohort of phenotypes A, B and C was 243 (20·0%), 458 (37·6%), and 516 patients (42·4%), respectively; and in the FEN-AUREUS cohort: 283 (23·9%), 573 (48·3%) and 329 (27·8%), respectively (Supplementary Tables S8 and S9).

### 30-Day mortality in the phenotypes and sub-phenotypes of the external validation cohorts

In the external validation cohorts, INSTINCT and FEN-AUREUS, the three phenotypes identified significant differences in 30-day mortality rates: 15·2% and 24·4% for phenotype A; 13·3% and 16·9% for phenotype B; and 26·9% and 28·6% for phenotype C, respectively (Table 2 and Supplementary Figures S2 and S3).

Furthermore, significant differences in 30-day mortality were also noted among the sub-phenotypes within each of the three phenotypes in both cohorts (Table 2), confirming the results of the derivation cohort. The aHRs for mortality were: 1·93 (95% CI: 1·01–3·71, p = 0·04) for sub-phenotype A2 vs A1; 3·40 (95% CI: 2·02–5·72, p < 0·0001) for B2 vs B1; and 3·04 (95% CI: 1·84–5·02, p < 0·0001) for C2 vs C1 in the INSTINCT cohort (Supplementary Table S10). In the FEN-AUREUS cohort, the corresponding aHRs were: 2·02 (95% CI: 1·25–3·29, p = 0·004); 2·11 (95% CI: 1·30–3·44, p = 0·002) and 2·44 (95% CI: 1·47–4·06) (Supplementary Table S11).

## Discussion

SAB is a condition characterised by significant heterogeneity, making the establishment of standardised management protocols highly complex.^7^^,^^8^ In this study, we identified three phenotypes of SAB based on probable portals of entry and six sub-phenotypes (two within each phenotype), taking into account baseline patient and infection characteristics, including demographics, comorbidities, acquisition type, site of infection, antibiotic susceptibility, and clinical and laboratory data from an international cohort. Both phenotypes and sub-phenotypes were associated with 30-day mortality. These phenotypes could provide an explanation for this heterogeneity and support the identification and management of these patients.

To our knowledge, only one previous study^15^ has aimed to identify clinical subphenotypes in SAB patients, using latent class analysis in both observational and trial cohorts. That study confirmed the existence of distinct, prognostically relevant groups. However, key methodological differences limit comparability. Swets et al. selected variables based on expert consensus and modelled class structure using both statistical and clinical criteria, whereas we applied an unsupervised, fully data-driven approach without preselection of variables. Their analyses focused primarily on trial populations, while our study was grounded in large, real-world cohorts with standardised data collection. Additionally, while Swets et al. suggest the possibility of differential treatment responses, our focus was on early clinical risk stratification, externally validated and operationalised through an open-access tool for routine application.

A recent study by Russell et al.^16^ highlighted distinct clinical trajectories in SAB, including early death, metastatic infection, and endocarditis. Although not based on clustering methods, their findings align with our phenotype-based approach in recognising meaningful patient subgroups with different clinical implications.

Interestingly, we were surprised to observe that the initial unsupervised clustering identified three phenotypes solely based on portals of entry, a variable often overlooked and/or misinterpreted in previous studies. A study by Del Rio et al.^17^ highlighted that approximately half of SAB cases lack a documented portal of entry, which significantly increases the risk of complications such as endocarditis and metastatic infections, as well as a poorer prognosis. In the ISAC cohort, the portal of entry and the site of infection were deliberately collected as separate variables—even though they sometimes overlapped—to acknowledge that, while these concepts may occasionally coincide, they are not synonymous and carry distinct clinical implications. Interestingly, a previous study found an association between certain MRSA clones and specific portals of entry,^18^ suggesting that entering the bloodstream via certain pathways would be facilitated by clone-related features. Thus, the ‘portal of entry’ variable holds important pathophysiological significance, as it reflects the route through which the pathogen enters the bloodstream.

Within each phenotype based on the portals of entry, two sub-phenotypes with different mortality were identified, which also varied in variables such as acquisition type, comorbidities, antibiotic susceptibility and laboratory data. The increased risk of patients according to the sub-phenotypes was confirmed in the validation cohorts. As expected, the characteristics of patients within the sub-phenotypes are not immediately evident due to the large number of variables used for their derivation. Consequently, multivariable models were developed to simplify the assignment of patients to their respective sub-phenotypes.

It is also worth noting that, in the analysis of the impact of sub-phenotypes on outcomes, consultation with infectious diseases specialists was associated with a lower hazard of mortality in both the ISAC and FEN-AUREUS cohorts. This finding aligns with previous studies^1^^,^^19^ and inherently highlights the critical importance of timely and effective treatment, as well as the vital role of these specialists in managing such infections. Additionally, early source control had a protective effect on mortality across all cohorts, further corroborating prior studies.^6^^,^^20^

The potential clinical applications of phenotype and sub-phenotype identification go beyond identifying patients at higher risk of mortality. For instance, they may be used to guide inclusion criteria or stratification in randomised controlled trials, potentially improving the precision and interpretation of trial outcomes by accounting for SAB heterogeneity. Moreover, future analyses will evaluate other clinically meaningful outcomes — including the occurrence of metastatic infections, the need for additional imaging, and the duration of therapy — in relation to the identified phenotypes and sub-phenotypes.

Some limitations of this study include the consideration that the probable portal of entry was determined by the treating clinician, though predefined criteria were used to minimise subjectivity. Inter-rater agreement could not be assessed due to the lack of independent classification by multiple clinicians, although the consistent reproducibility of phenotypes across cohorts and the use of standardised definitions likely mitigated variability. The analysis of sub-phenotypes was not explicitly predefined in the original protocol but was conducted post hoc to explore additional heterogeneity within the identified phenotypes. We did not include host genetic or pathophysiological analyses, but these are planned as part of the broader FEN-AUREUS study protocol (Supplementary Material). Ongoing sample collection will enable further exploration of microbiological, immunological, and genetic underpinnings of the identified phenotypes and sub-phenotypes. Additionally, eosinophil counts and data on race or ethnicity were not systematically collected in the original cohorts, which should be considered when interpreting the findings.

The strengths of the study include the use of well-characterized, large-scale international cohorts, the inclusion of a high number of clinical variables, and the external validations conducted. Additionally, to facilitate clinical applicability, a freely accessible online calculator for phenotypes and sub-phenotypes assignment was developed (https://fen-aureus.com/).

In conclusion, hospitalized patients with SAB can be classified into phenotypes and sub-phenotypes with different mortality risk. An algorithm based on a simplified tool for the probabilistic classification of patients into phenotypes and sub-phenotypes was proposed. Further studies are needed to elucidate the underlying pathophysiological mechanisms leading to specific phenotypes and sub-phenotypes, as well as to explore other potential clinical applications. Our findings aim to provide a practical tool for clinicians, facilitating more personalized care for patients with SAB.

## Contributors

Study conception and design: BGG, JRB. Acquisition of data: all members of the FEN-AUREUS/ISAC/INSTINCT groups. Analyses and interpretation of data: BGG, JRB, BGM. Manuscript draft: BGG, JRB, BGM. Manuscript critical revision all members of the FEN-AUREUS/ISAC/INSTINCT groups. Obtaining funds: BGG, JRB. All authors had access to the data; BGG and JR-B verified all data and were responsible for the decision to submit the manuscript.

## Data sharing statement

Data collected for the study, including deidentified participant data and a data dictionary defining each field in the set, will be made available to other investigators upon request to the corresponding author, after approval of a proposal by the FISEVI–FEN-AUREUS–ISAC–INSTINCT groups boards, with a signed data access agreement, beginning with publication.

## Declaration of interests

Daniel Hornuss reports honoraria from Pfizer, Streamed Up, and Thieme Publications.

Siegbert Rieg reports honoraria for lectures from Falk Foundation, Pfizer, bioMérieux, and other medical education providers, including Akademie für Infektionsmedizin, Med Update GmbH, streamedup! GmbH, Deutsches Beratungszentrum für Hygiene, Deutscher Apotheker-Verlag, Forum für medizinische Fortbildung, and Meet The Experts Academy.

Esperanza Merino de Lucas reports grants from Instituto de Salud Carlos III; honoraria for lectures from MSD, Tillots, Gilead, Menarini, AstraZeneca, and Angelini; support for travel from MSD and Gilead; and participation on advisory boards for Tillots and Advanz Pharma.

Antonio Plata-Ciézar reports honoraria from Menarini, Pfizer, and Shionogi, and participation on advisory boards for Shionogi and Menarini.

Luis Eduardo López-Cortés reports consulting fees from Angelini (scientific advisor), and honoraria for lectures from Angelini, ViiV, Gilead, and Correvio.

All other authors declare no potential conflicts of interest.

## Appendix A Supplementary data

Supplementary Material **and** Protocol

Supplementary ISAC Study Group author list

Supplementary INSTINCT study group author list

Supplementary FEN-AUREUS study group author list
