# Supplementary material for: Identification and validation of clinical phenotypes in Staphylococcus aureus bloodstream infection and their association with mortality (FEN-AUREUS study)
Source: eClinicalMedicine. 2025 May 7;83:103240. doi: 10.1016/j.eclinm.2025.103240 (PMC12235390; doi:10.1016/j.eclinm.2025.103240)
Supplement: Supplementary Material and Protocol [file mmc1.pdf]

SUPPLEMENTARY MATERIAL- APPENDIX

Table of contents

- Table S1. Common definitions of different probable portals of entry and sources of infection: **p. 2**
- Table S2. Missing data for the variables collected in the derivation cohort (ISAC) with missing data >1%: **p. 3**
- Table S3. Features and outcomes of patients with A phenotype (portal of entry: skin and soft tissue) in the derivation cohort, based on identified sub-phenotypes.: **pp. 4–6**
- Table S4. Features and outcomes of patients with B phenotype (portal of entry: vascular catheter) in the derivation cohort, based on identified sub-phenotypes.: **pp. 7–9**
- Table S5. Features and outcomes of patients with C phenotype (portal of entry: other/unknown) in the derivation cohort, based on identified sub-phenotypes.: **pp. 10–12**
- Table S6**.** Multivariable models to predict sub-phenotype 2 in each of the previously identified phenotypes based on the portal of entry.: **p. 13**
- Table S7. Distribution of the phenotypes identified in the cluster analysis in the INSTINCT cohort based on the portal of entry.: **p.14**
- Figure S1. Heatmap of variable deviations from the mean across sub-phenotypes.: **p. 15–16**
- Table S8. Features and outcomes of patients in the INSTINCT external validation cohort and in the phenotypes obtained.: **pp. 17–18**
- Table S9. Features and outcomes of patients in the FEN-AUREUS external validation cohort and in the phenotypes obtained**.: pp. 19–20**
- Figure S2. Kaplan-Meier survival analysis: 30-day mortality by phenotype and sub-phenotype in the INSTINCT external validation cohort.: **pp. 21–22**
- Table S10. Multivariate Cox regression of 30-day mortality for each of the identified phenotypes in the INSTINCT cohort.: **p.23**
- Figure S3**.** Kaplan-Meier survival analysis: 30-day mortality by phenotype and sub-phenotype in the FEN-AUREUS external validation cohort.: **pp. 24–25**
- Table S11. Multivariate Cox regression of 30-day mortality for each of the identified phenotypes in the FEN-AUREUS cohort**.: p. 26**
- Study protocol**.: p. 27**

Table S1. Common definitions of different probable portals of entry and sources of infection

Probable portal of entry	Definition
Skin and soft tissue	Clinically apparent infection unrelated to surgical intervention, infected surgical wound with or without an associated prosthesis.
Vascular catheter-related	Isolation of *S. aureus* from both the catheter tip and blood with identical susceptibility profiles, or a differential time to positivity of more than two hours between blood cultures from a peripheral vein and the catheter.
Respiratory tract	Includes ventilator-associated pneumonia, hospital-acquired pneumonia, and community-acquired pneumonia.
Genito-urinary tract	Infections related to per-urethral or supra-pubic urinary catheters, or recent urological or gynaecological/obstetric surgery within one week of bacteraemia.
Unknown	No probable portal could be determined based on available evidence.

Site of infection	Definition
Central venous line	Infection at the site of a central venous line, including PICC line.
Peripheral venous line	Infection at the site of a peripheral venous line.
Surgical wound	Infection at the site of a surgical wound.
Skin/Soft tissue (excluding wounds)	Infection in the skin or soft tissue not related to a surgical wound.
Lung	Infection in the lungs, such as pneumonia.
Genito-urinary tract	Infection in the urinary tract, including infections related to urinary catheters or recent urological surgery.
Native heart valve	Infection involving the native heart valve.
Prosthetic heart valve	Infection involving a prosthetic heart valve.
Implanted vascular device	Infection related to an implanted vascular device, such as a pacemaker, stent, or graft.
Infected intravascular thrombus	Infection associated with an intravascular thrombus.
Vertebral bone/disc	Infection involving the vertebral bones or intervertebral discs.
Epidural or intraspinal empyema	Infection involving the epidural space or spinal cord.
Native joint	Infection in a native joint.
Prosthetic joint	Infection in a prosthetic joint.
Other bone	Infection in other bones not specified elsewhere.
Deep tissue infection or abscess	Infection involving deep tissues or abscesses, such as psoas or splenic abscess.
Other	Any other site of infection not specified above.
Foci not established	The site of infection could not be identified after thorough diagnostic evaluation.

Table S2. Missing data for the variables collected in the derivation cohort (ISAC) with missing data >1%.

**Variables**	**% Missing data**
Intravenous drug use	2·9%
Peritoneal dialysis	1·1%
Hemodialysis	1·2%
Systemic corticosteroids more 2 weeks	1·3%
Neutropenia	1·5%
Chemotherapy current	1·4%
Inmunesuppresive therapy	2·0%
Organ or bone marrow transplantation	1·1%
HIV	8·5%
Catheter at onset	5·3%
O2 saturation	21·4%
FiO2	30·1%
Lowest Glasgow score	11·8%
Serum bilirubin	33·2%
Platelet count	7·0%
Serum creatinine	9·8%
Maximum temperature	13·2%
Minimum temperature	21·4%
Maximum respiratory rate	31·0%
Maximum heart rate	16·0%
Serum CRP Closest to First Positive Culture	17·8%
Peripheral Blood White Cell Count	6·9%
Neutrophil Count	25·9%
Lymphocyte Count	32·1%

Table S3. Features and outcomes of patients with A phenotype in the derivation cohort, based on identified sub-phenotypes.

Factor	A1 (n= 370)	A2 (n=88)	p.value
**Demographics**			
Female sex	121 (32·7)	34 (38·6)	0·32
Median age in years (IQR)	65 (52–77)	66 (54–78)	0·52
**Comorbidities**			
Myocardial infarction	57 (15·4)	20 (22·7)	0·11
Peripheral vascular disease	51 (13·8)	22 (25·0)	0·01
Dementia	28 (7·6)	15 (17·0)	0·01
Chronic lung disease	43 (11·6)	11 (12·5)	0·85
Leukaemia	9 (2·4)	4 (4·5)	0·29
Lymphoma	10 (2·7)	3 (3·4)	0·72
Tumour without metastasis	31 (8·4)	5 (5·7)	0·51
Metastatic solid tumour	11 (3·0)	5 (5·7)	0·21
Chronic renal disease	53 (14·3)	47 (53·4)	<0·001
Cerebrovascular disease			0·48
No cerebrovascular disease	316 (85·4)	71 (80·7)	
Mild or no residual neurological defect	37 (10·0)	12 (13·6)	
Hemiplegia	17 (4·6)	5 (5·7)	
Chronic liver disease			0·03
No liver disease	316 (85·4)	81 (92·0)	
Mild	32 (8·6)	1 (1·1)	
Moderate or severe	22 (5·9)	6 (6·8)	
Diabetes mellitus			<0·001
Not diabetic	242 (65·4)	36 (40·9)	
Without end-organ damage	76 (20·5)	19 (21·6)	
With end-organ damage	52 (14·1)	33 (37·5)	
HIV infection	7 (1·9)	2 (2·3)	0·68
**Invasive procedures or treatment**			
Hemodialysis	9 (2·4)	28 (31·8)	<0·001
Systemic corticosteroid for more than 2 weeks	19 (5·1)	7 (8·0)	0·31
Neutropenia	2 (0·5)	0 (0·0)	>0·99
Current chemotherapy	0 (0·0)	5 (5·7)	<0·001
Immune suppressive therapy	23 (6·2)	4 (4·5)	0·80
Organ or bone marrow transplantation	9 (2·4)	5 (5·7)	0·16
Previous surgery	4 (1·1)	0 (0·0)	>0·99
Vascular catheter at onset	62 (16·8)	15 (17·0)	>0·99
**Acquisition of infection**			
Community-acquired	159 (43·0)	22 (25·0)	0·002
Healthcare associated	110 (29·7)	42 (47·7)	0·002

Nosocomial	102 (27·6)	24 (27·3)	>0·99
**Specific likely portal of entry**			
Infection-unrelated surgical wound	253 (68·4)	71 (80·7)	0·02
Infection-related surgical wound	117 (31·6)	17 (19·3)	
**Main site of infection**[1]			
Skin soft tissue excluding surgical wound and deep tissue	156 (42·2)	36 (40·9)	0·90
Surgical wound	72 (19·5)	16 (18·2)	0·88
Central venous catheter (including PICC)	5 (1·4)	2 (2·3)	0·62
Peripheral venous catheter	8 (2·2)	3 (3·5)	0·46
Infected intravascular thrombus	7 (1·9)	3 (3·4)	0·41
Implanted vascular device	2 (0·5)	1 (1·1)	0·47
Native heart valve	18 (4·9)	3 (3·4)	0·78
Prosthetic heart valve	5 (1·4)	4 (4·5)	0·07
Epidural or intraspinal empyema	5 (1·4)	0 (0·0)	0·59
Vertebral bone/disc	17 (4·6)	1 (1·1)	0·22
Native joint	24 (6·5)	9 (10·2)	0·25
Prosthetic joint	10 (2·7)	3 (3·4)	0·72
Other bone-related source	30 (8·1)	12 (13·6)	0·15
Deep tissue infection or abscess	42 (11·4)	7 (8·0)	0·44
Pneumonia	22 (5·9)	6 (6·8)	0·80
Source not established	22 (5·9)	3 (3·4)	0·44
Other sources	13 (3·5)	7 (8·0)	0·08
**Other Infection features**			
Previous non-bacteraemic *S. aureus* infection in the last 12 weeks	17 (4·6)	1 (1·1)	0·22
More than 2 days with symptoms	114 (30·8)	30 (34·1)	0·61
Median days from admission until confirmation of *S. aureus* bacteraemia (IQR)	0 (0–4)	0 (0–7)	0·16
**Antibiotic susceptibility**			
Methicillin-resistant (%)	67 (18·1)	38 (43·2)	<0·001
**Physical examination data**			
Hypotension (arterial blood pressure < 70mmHG)	66 (17·8)	30 (34·1)	0·001
Inotropes use	39 (10·5)	20 (22·7)	0·004
Lowest Glasgow score (IQR)	15 (14–15)	15 (12–15)	0·003
Highest temperature (ºC), median (IQR)	38·3 (37·5–38·9)	38·0 (37·4–38·6)	0·19
Maximum heart rate (bpm), median (IQR)	98 (88–110)	104 (92–120)	0·002
**Laboratory analysis**			
Lowest O2 saturation in %, median (IQR)	94 (93–96)	93 (90–95)	0·005
Highest FiO2 %, median (IQR)	21 (21–34)	29 (21–43)	0·004
Serum bilirubin (mg/dL), median (IQR)	0·70 (0·50–1·00)	0·40 (0·25–0·60)	0·001
Serum creatinine (mg/dL), median (IQR)	1·16 (0·81–1·63)	2·32 (1·23–5·36)	<0·001

Serum CRP (mg/L), median (IQR)	156·0 (65·0–230·0)	142·5 (38·8–242·0)	0·31
Blood white cell count (x10^3/μL), median (IQR)	12·9 (8·9–16·4)	13·6 (9·9–19·6)	0·11
Neutrophil count (x10^3/μL), median (IQR)	10·8 (7·3–13·9)	12·1 (8·6–19·2)	0·006
Lymphocyte count (x10^3/μL), median (IQR)	1·2 (0·7–1·7)	1·1 (0·6–1·6)	0·67
Platelet count (x10^3/μL), median (IQR)	215 (140–297)	239 (165–300)	0·29

[1] More than one allowed

Table S4. Features and outcomes of patients with B phenotype in the derivation cohort, based on identified sub-phenotypes.

Factor	B1 (n=498)	B2 (n=75)	P value
**Demographics**			
Female sex	184 (36·9)	28 (37·3)	>0·99
Median age in years (IQR)	62 (50–73)	65 (52–73)	0·57
**Comorbidities**			
Myocardial Infarction	89 (17·9)	7 (9·3)	0·07
Peripheral vascular disease	45 (9·0)	10 (13·3)	0·29
Dementia	15 (3·0)	4 (5·3)	0·29
Chronic lung disease	74 (14·9)	17 (22·7)	0·09
Leukaemia	17 (3·4)	5 (6·7)	0·19
Lymphoma	32 (6·4)	3 (4·0)	0·60
Tumour without metastasis	42 (8·4)	12 (16·0)	0·05
Metastatic solid tumour	103 (20·7)	18 (24·0)	0·54
Chronic renal disease	179 (35·9)	22 (29·3)	0·3
Cerebrovascular disease			0·01
No cerebrovascular disease	433 (86·9)	59 (78·7)	
Mild or no residual neurological defect	50 (10·0)	8 (10·7)	
Hemiplegia	15 (3·0)	8 (10·7)	
Chronic liver disease			0·81
No liver disease	449 (90·2)	67 (89·3)	
Mild	25 (5·0)	3 (4·0)	
Moderate or severe	24 (4·8)	5 (6·7)	
Diabetes mellitus			<0·001
Not diabetic	348 (69·9)	49 (65·3)	
Without end-organ damage	67 (13·5)	25 (33·3)	
With end-organ damage	83 (16·7)	1 (1·3)	
HIV infection	4 (0·8)	2 (2·7)	0·18
**Invasive procedures or treatment**			
Hemodialysis	121 (24·3)	13 (17·3)	0·24
Systemic corticosteroid for more than 2 weeks	45 (9·0)	9 (12·0)	0·40
Neutropenia	23 (4·6)	2 (2·7)	0·76
Current Chemotherapy	76 (15·3)	8 (10·7)	0·38
Immune suppressive therapy	32 (6·4)	6 (8·0)	0·62
Organ or bone marrow transplantation	21 (4·2)	3 (4·0)	>0·99
Previous surgery	0 (0·0)	1 (1·3)	0·13
Vascular catheter at onset	402 (80·7)	55 (73·3)	0·16
**Acquisition**			
Community Acquired	25 (5·0)	5 (6·7)	0·58

Healthcare Associated	183 (36·7)	17 (22·7)	0·02
Nosocomial	290 (58·2)	53 (70·7)	0·04
**Specific likely portal of entry**			
Peripheral IV Catheter	183 (36·7)	23 (30·7)	0·37
Central intravenous catheter	219 (44·0)	52 (69·3)	<0·001
Other implanted vascular device	96 (19·3)	0 (0·0)	<0·001
**Main site of infection**[1]			
Skin and soft tissues excluding surgical wound and deep tissue	17 (3·4)	0 (0·0)	0·15
Surgical wound	6 (1·2)	1 (1·3)	>0·99
Central venous catheter (including PICC)	210 (42·2)	42 (56·0)	0·03
Peripheral venous catheter	164 (32·9)	18 (24·0)	0·14
Infected intravascular thrombus	12 (2·4)	2 (2·7)	0·70
Implanted vascular device	91 (18·3)	4 (5·3)	0·004
Native heart valve	15 (3·0)	4 (5·3)	0·29
Prosthetic heart valve	3 (0·6)	1 (1·3)	0·43
Epidural or intraspinal empyema	2 (0·4)	2 (2·7)	0·09
Vertebral bone/disc	3 (0·6)	4 (5·3)	0·007
Native joint	8 (1·6)	3 (4·0)	0·16
Prosthetic joint	2 (0·4)	1 (1·3)	0·34
Other bone-related source	1 (0·2)	0 (0·0)	>0·99
Deep tissue infection or abscess	5 (1·0)	1 (1·3)	0·57
Pneumonia	8 (1·6)	1 (1·3)	>0·99
Source not established	12 (2·4)	5 (6·7)	0·06
Other sources	23 (4·6)	4 (5·3)	0·77
**Other Infection features**			
Previous non-bacteraemic *S. aureus* infection in the last 12 weeks	7 (1·4)	14 (18·7)	<0·001
More than 2 days with symptoms	63 (12·7)	11 (14·7)	0·58
Median days from admission until confirmation of *S. aureus* bacteraemia	4·00 (0·00–12·00)	8·50 (1·00–30·50)	0·003
**Antibiotic susceptibility**			
Methicillin-resistant	54 (10·8)	40 (53·3)	<0·001
**Physical examination data**			
Hypotension (arterial blood pressure<70mmHG)	87 (17·5)	24 (32·0)	0·005
Inotropes use	37 (7·4)	19 (25·3)	<0·001
Lowest Glasgow score (IQR)	15 (15–15)	14 (9–15)	<0·001
Highest temperature (ºC), median (IQR)	38·5 (38·1–39·0)	38·4 (37·5–38·9)	0·009
Maximum heart rate (bpm), median (IQR)	98 (90–110)	106 (92–127)	0·001
**Laboratory data**			
Lowest O2 saturation in %, median (IQR)	95 (94–97)	93 (90–96)	<0·001
Highest FiO2 %, median (IQR)	24 (21–34)	31 (22–45)	<0·001
Serum bilirubin (mg/dL), median (IQR)	0·77 (0·50–1·15)	0·70 (0·45–1·05)	0·43

Serum creatinine (mg/dL), median (IQR)	1·21 (0·80–3·76)	0·96 (0·69–2·84)	0·04
Serum CRP (mg/L), median (IQR)	106·1 (38·0–162·8)	84·0 (31·7–161·6)	0·23
Blood white cell count (x10^3/µL), median (IQR)	10·7 (7·0–14·1)	12·9 (8·2–17·5)	0·005
Neutrophil count (x10^3/µL), median (IQR)	9·0 (6·0–12·1)	11·9 (7·2–17·2)	<0·001
Lymphocyte count (x10^3/µL), median (IQR)	0·85 (0·50–1·30)	0·80 (0·45–1·20)	0·68
Platelet count (x10^3/µL), median (IQR)	185 (123–252)	191 (123–259)	0·84

[1] More than one allowed

Table S5. Features and outcomes of patients with C phenotype in the derivation cohort, based on identified sub-phenotypes.

Factor	C1 (n=403)	C2 (n=694)	P value
**Demographics**			
Female sex	158 (39·2)	252 (36·3)	0·36
Median age in years (IQR)	61 (48–75)	70 (59–79)	<0·001
**Comorbidities**			
Myocardial Infarction	33 (8·2)	114 (16·4)	<0·001
Peripheral vascular disease	16 (4·0)	73 (10·5)	<0·001
Dementia	19 (4·7)	65 (9·4)	0·005
Chronic lung disease	38 (9·4)	112 (16·1)	0·002
Leukaemia	10 (2·5)	16 (2·3)	0·84
Lymphoma	10 (2·5)	21 (3·0)	0·71
Solid cancer without metastasis	24 (6·0)	75 (10·8)	0·006
Metastatic solid cancer	44 (10·9)	52 (7·5)	0·06
Chronic renal disease	39 (9·7)	189 (27·2)	<0·001
Cerebrovascular disease			<0·001
No cerebrovascular disease	380 (94·3)	562 (81·0)	
Mild or no residual neurological defect	16 (4·0)	89 (12·8)	
Hemiplegia	7 (1·7)	43 (6·2)	
Chronic liver disease			<0·001
No liver disease	326 (80·9)	590 (85·0)	
Mild	47 (11·7)	33 (4·8)	
Moderate or severe	30 (7·4)	71 (10·2)	
Diabetes mellitus			<0·001
Not diabetic	342 (84·9)	458 (66·0)	
Without end-organ damage	49 (12·2)	142 (20·5)	
With end-organ damage	12 (3·0)	94 (13·5)	
HIV infection	9 (2·2)	6 (0·9)	0·10
**Invasive procedures or treatment**			
Hemodialysis	3 (0·7)	64 (9·2)	<0·001
Systemic corticosteroid for more than 2 weeks	16 (4·0)	47 (6·8)	0·06
Neutropenia	11 (2·7)	24 (3·5)	0·59
Current chemotherapy	35 (8·7)	43 (6·2)	0·14
Immunesuppressive therapy	24 (6·0)	34 (4·9)	0·48
Organ or bone marrow transplantation	14 (3·5)	29 (4·2)	0·63
Previous surgery (last month)	2 (0·5)	1 (0·1)	0·56
Vascular catheter at onset	73 (18·1)	225 (32·4)	<0·001
**Acquisition of infection**			
Community Acquired	217 (53·8)	254 (36·6)	<0·001

Healthcare Associated	147 (36·5)	127 (18·3)	<0·001
Nosocomial	39 (9·7)	313 (45·1)	<0·001
**Likely portal of entry**			
Respiratory	42 (10·4)	111 (16·0)	0·01
Genito-Urinary Tract	93 (23·1)	86 (12·4)	<0·001
Portal not established	268 (66·5)	497 (71·6)	0·07
**Main site of infection** [1]			
Skin and soft tissues excluding surgical wound and deep tissue	14 (3·5)	25 (3·6)	>0·99
Surgical wound	0	0	-
Central venous catheter (including PICC)	0	0	-
Peripheral venous catheter	0	0	-
Infected intravascular thrombus	7 (1·7)	4 (0·6)	0·11
Implanted vascular device	15 (3·7)	27 (3·9)	0·89
Native heart valve	47 (11·7)	35 (5·)	<0·001
Prosthetic heart valve	12 (3·0)	15 (2·2)	0·40
Epidural or intraspinal empyema	29 (7·2)	8 (1·2)	<0·001
Vertebral bone/disc	67 (16·6)	20 (2·9)	<0·001
Native joint	59 (14·6)	17(2·4)	<0·001
Prosthetic joint	9 (2·2)	19 (2·7)	0·69
Other bone-related source	13 (3·2)	7 (1·0)	0·02
Deep tissue infection or abscess	69 (17·1)	31 (4·5)	<0·001
Pneumonia	41 (10·2)	139 (20·0)	<0·001
Source not established	66 (16·4)	296 (42·6)	<0·001
Other sources	15 (3·7)	29 (4·2)	0·71
**Other Infection features**			
Previous non-bacteraemic *S. aureus* infection in the last 12 weeks	7 (1·7)	51 (7·3)	<0·001
More than 2 days with symptoms	142 (35·2)	113 (16·3)	<0·001
Median days from admission until confirmation of *S. aureus* bacteraemia (IQR)	0 (0–1)	1 (0–7)	<0·001
**Antibiotic susceptibility**			
Methicillin-resistant	18 (4·5)	157 (22·6)	<0·002
**Physical examination data**			
Hypotension (arterial blood pressure<70mmHG)	74 (18·4)	162 (23·3)	0·06
Inotropes use	44 (10·9)	135 (19·5)	<0·001
Lowest Glasgow score (IQR)	15 (14–15)	15 (13–15)	<0·001
Highest temperature (ºC), median (IQR)	38·2 (37·5–38·6)	38·2 (37·5–38·70)	0·61
Highest heart rate (bpm), median (IQR)	98 (90–110)	99 (89–117)	0·36
**Laboratory analysis**			
Lowest O2 saturation in %, median (IQR)	95 (93–96)	94 (91–96)	0·007
Highest FiO2 %, median (IQR)	24 (21–33)	30 (21–41)	<0·001
Serum bilirubin (mg/dL), median (IQR)	0·68 (0·43–1·05)	0·72 (0·45–1·10)	0·36

Serum creatinine (mg/dL), median (IQR)	1·03 (0·75–1·37)	1·31 (0·81–2·50)	<0·001
Serum CRP (mg/L), median (IQR)	136·0 (44·0–204·2)	103·0 (34·2–185·6)	0·003
Blood white cell count (x10^3/µL), median (IQR)	13·0 (9·0–16·2)	12·5 (8·3–17·4)	0·70
Neutrophil count (x10^3/µL), median (IQR)	10·8 (7·4–13·8)	10·5 (7·1–15·1)	0·77
Lymphocyte count (x10^3/µL), median (IQR)	0·90 (0·50–1·30)	1·00 (0·60–1·40)	0·25
Platelet count (x10^3/µL), median (IQR)	197 (125–266)	185 (107–251)	0·03

[1] More than one allowed

Table S6. Multivariable models to predict sub-phenotype 2 in each of the previously identified phenotypes based on the portal of entry.

VARIABLE	PHENOTYPE					
	A[1]		B[2]		C[3]	
	aOR (95% CI)	p	aOR (95% CI)	p	aOR (95% CI)	p
Age, per unit					1·02 (1·01–1·03)	<0·0001
Peripheral vascular disease	1·88 (0·93–3·80)	0·08				
Dementia	2·78 (1·13–6·82)	0·03				
Chronic renal disease	3·25 (1·62–6·54)	0·0009			1·58 (0·97–2·57)	0·06
Congestive heart failure					1·65 (1·07–2·54)	0·04
Any tumour			2·04 (0·99–4·20)	0·05		
Diabetes mellitus without end-organ damage					2·14 (1·35–3·39)	0·001
Diabetes mellitus with end –organ damage					3·63 (1·65–7·99)	0·0006
Previous non-bacteraemic *S· aureus* infection in the last 12 weeks			24·9 (8·02–77·60)	<0·0001	7·84 (3·31–18·01)	<0·0001
Nosocomial acquisition					11·59 (7·89–17·02)	<0·0001
Infection related surgical wound	0·42 (0·21–0·86)	0·02				
Deep tissue infection or abscess at the time first positive blood culture was taken					0·35 (0·22–0·56)	0·001
Pneumonia as main focus of infection at the time first positive blood culture was taken					1·77 (1·01–3·10)	0·05
Foci not established at the time first positive blood culture was taken					5·21 (4·19–6·48)	<0·0001
Methicillin-resistant *Staphylococcus aureus* (MRSA)	3·96 (2·13–7·39)	<0·0001	12·2 (6·53–22·90)	<0·0001	5·69 (2·85–11·36)	<0·0001
Oxygen saturation, room air, pulse oximetry (per percent point)			0·94 (0·91–0·97)	0·0008	0·97 (0·95–0·99)	0·04
Neutrophiles count (per 10^3 cells/µL)	1·03 (1·01–1·05)	0·002	1·06 (1·04–1·08)	<0·0001		
Glasgow Coma Scale (GCS) score at admission, per unit					0·90 (0·85–0·96)	<0·0001
Serum creatinine concentration at Day 0 (per 1 mg/dL)	1·46 (1·24–1·71)	<0·0001			1·43 (1·23–1·66)	<0·0001

The variance inflation factor value was less than 2 in all cases· OR=odds ratio.

[1] Model for sub-phenotype A2 among patients with A phenotype. AUROC (95% CI) of the model: 0·86 (0·82–0·91). The optimal threshold was 0·19, yielding a sensitivity of 76·0% and specificity of 82·0%. The positive predictive value (PPV) was 50·0%, the negative predictive value (NPV) was 93·5%, and the overall accuracy was 80·8%.

[2] Model for sub-phenotype B2 among patients with B phenotype. AUROC (95% CI) of the model: 0·88 (0·83–0·92). The optimal threshold was 0·10, yielding a sensitivity of 82·7% and specificity of 85·9%. The positive predictive value (PPV) was 47·0%, the negative predictive value (NPV) was 97·1%, and the overall accuracy was 85·5%.

[3] Model for sub-phenotype C2 among patients with C phenotype. AUROC (95% CI) of the model: 0·89 (0·87–0·91). The optimal threshold was 0·52, yielding a sensitivity of 88·6% and specificity of 75·2%. The positive predictive value (PPV) was 86·0%, the negative predictive value (NPV) was 79·3%, and the overall accuracy was 83·7%.

Table S7. Distribution of the phenotypes identified in the cluster analysis in the INSTINCT cohort based on the portal of entry.

Phenotype identified in cluster analysis	Portal of entry		
	Skin and soft tissues	Vascular catheter	Other or unknown
Ac (n=355)	**286 (80·5)**	24 (6·8)	45 (12·7)
Bc (n=432)	1 (0·2)	**431 (99·8)**	0
Cc (n=430)	18 (4·2)	3 (0·7)	**409 (95·1)**

Figure S1. Heatmap of variable deviations from the mean across sub-phenotypes

This figure shows heatmaps of deviations from the mean for categorical variables (S1.A) and continuous variables (S1.B) across different subphenotypes. For categorical variables, the percentage deviations were calculated by comparing the proportion of each variable in a given subphenotype to the overall mean proportion across all subphenotypes. For continuous variables, Z-scores were computed to represent how far each subphenotype's mean value deviates from the overall mean, standardised by the variable's standard deviation.

The colour scale ranges from dark blue, indicating the largest deviations below the mean, through grey, representing values near the mean, to dark red, which reflects the largest deviations above the mean. This approach allows for visual identification of patterns and variables that differentiate subphenotypes, highlighting potential clinical or epidemiological significance.

A

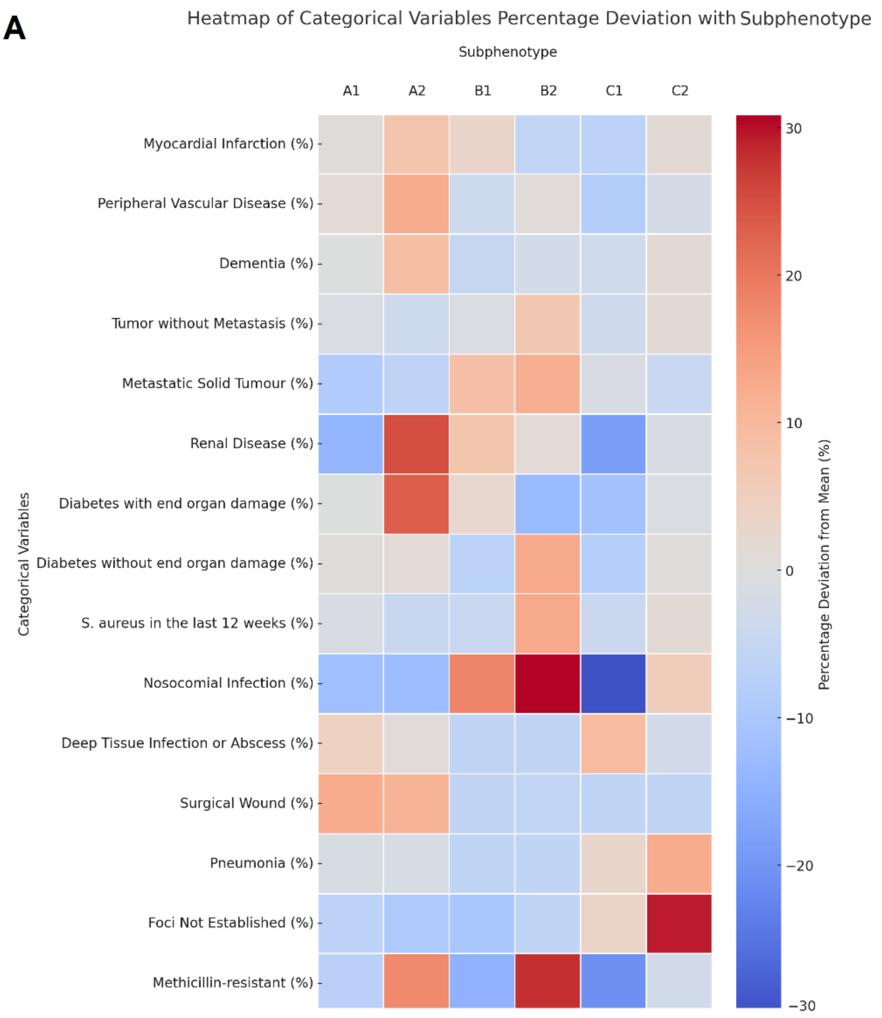

Heatmap of Categorical Variables Percentage Deviation with Subphenotype
Subphenotype
A1
A2
B1
B2
C1
C2
Myocardial Infarction (%)
Peripheral Vascular Disease (%)
Dementia (%)
Tumor without Metastasis (%)
Metastatic Solid Tumour (%)
Renal Disease (%)
Diabetes with end organ damage (%)
Diabetes without end organ damage (%)
S. aureus in the last 12 weeks (%)
Nosocomial Infection (%)
Deep Tissue Infection or Abscess (%)
Surgical Wound (%)
Pneumonia (%)
Foci Not Established (%)
Methicillin-resistant (%)
Categorical Variables
30
20
10
0
−10
−20
−30
Percentage Deviation from Mean (%)

B

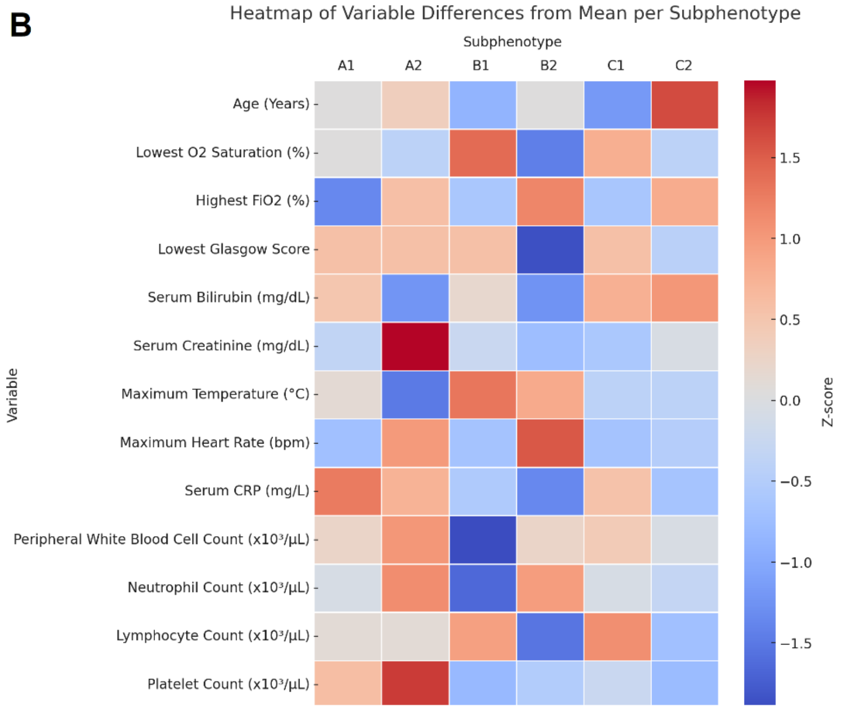

Heatmap of Variable Differences from Mean per Subphenotype
Subphenotype
A1
A2
B1
B2
C1
C2
Age (Years)
Lowest O2 Saturation (%)
Highest FiO2 (%)
Lowest Glasgow Score
Serum Bilirubin (mg/dL)
Serum Creatinine (mg/dL)
Maximum Temperature (°C)
Maximum Heart Rate (bpm)
Serum CRP (mg/L)
Peripheral White Blood Cell Count (x10³/μL)
Neutrophil Count (x10³/μL)
Lymphocyte Count (x10³/μL)
Platelet Count (x10³/μL)
Variable
1.5
1.0
0.5
0.0
−0.5
−1.0
−1.5
Z-score

Table S8. Features and outcomes of patients in the INSTINCT external validation cohort and in the phenotypes obtained.

Factor	A phenotype (n=243)	B phenotype (n=458)	C phenotype (n=516)	P value
**Demographics**				
Female sex	86 (35·4)	141 (31·7)	177 (34·3)	0·54
Median age in decades (IQR)	70 (60–80)	70 (50–70)	70 (50–80)	0·27
**Comorbidities**				
Congestive heart failure	55 (22·6)	83 (18·1)	94 (18·2)	0·28
Peripheral vascular disease	35 (14·4)	39 (8·5)	33 (6·4)	0·001
Dementia	5 (2·1)	16 (3·5)	19 (3·7)	0·48
Chronic Lung Disease	28 (11·5)	55 (12·0)	61 (11·8)	0·98
Tumour	31 (12·8)	131 (28·6)	85 (16·5)	<0·001
Chronic renal Disease	68 (28·0)	147 (32·1)	124 (24·0)	0·02
Cerebrovascular Disease	32 (13·2)	82 (17·9)	70 (13·6)	0·11
Chronic liver disease				0·001
No liver disease	214 (88·1)	408 (89·1)	415 (80·4)	
Mild	16 (6·6)	24 (5·2)	41 (7·9)	
Moderate or severe	13 (5·3)	26 (5·7)	60 (11·6)	
Diabetes mellitus	92 (37·9)	105 (22·9)	118 (22·9)	<0·001
HIV Infection	2 (0·8)	8 (1·7)	10 (1·9)	0·52
**Invasive procedures or treatment**				
Neutropenia	15 (6·2)	65 (14·2)	43 (8·3)	0·001
Current chemotherapy	63 (25·9)	98 (21·4)	147 (28·5)	0·04
Immunesuppressive therapy	21 (8·6)	47 (10·3)	37 (7·2)	0·23
Vascular catheter at onset	30 (12·3)	97 (21·2)	48 (9·3)	<0·001
**Acquisition of infection**				
Community Acquired	34 (14·0)	4 (0·9)	173 (33·5)	<0·001
Healthcare Associated	94 (38·7)	116 (25·3)	139 (26·9)	<0·001
Nosocomial Infection	115 (47·3)	338 (73·8)	204 (39·5)	<0·001
**Main site of infection at the time first blood culture was taken[1]**				
Skin and soft tissues excluding surgical wound and deep tissue	63 (25·9)	15 (3·3)	1 (0·2)	<0·001
Surgical wound	51 (21·0)	0 (0·0)	0 (0·0)	<0·001
Central venous catheter (including PICC)	0 (0·0)	81 (17·7)	0 (0·0)	<0·001
Peripheral venous catheter	0 (0·0)	127 (27·7)	0 (0·0)	<0·001
Infected intravascular thrombus	0 (0·0)	9 (2·0)	1 (0·2)	<0·001
Implanted vascular device	4 (1·6)	161 (35·1)	13 (2·5)	<0·001
Native heart valve	17 (7·0)	19 (4·1)	67 (13·0)	<0·001
Prosthetic heart valve	4 (1·6)	7 (1·5)	13 (2·5)	0·50
Epidural or intraspinal empyema	2 (0·8)	0 (0·0)	7 (1·4)	0·05
Vertebral bone/disc	46 (18·9)	5 (1·1)	62 (12·0)	<0·001
Native joint	4 (1·6)	4 (0·9)	19 (3·7)	0·01
Prosthetic joint	6 (2·5)	1 (0·2)	10 (1·9)	0·02

Other bone-related source	29 (11·9)	1 (0·2)	4 (0·8)	<0·001
Deep tissue infection or abscess	4 (1·6)	3 (0·6)	19 (3·7)	0·005
Pneumonia	5 (2)	3 (0·7)	68 (13·2)	<0·001
Source not established	23 (9·5)	11 (2·4)	193 (37·4)	<0·001
Other sources	12 (5·0)	11 (2·4)	45 (8·7)	<0·001
**Medical history**				
Previous non-bacteraemic *S. aureus* infection in the last 12 weeks	2 (0·8)	12 (2·6)	10 (1·9)	0·26
**Antibiotic susceptibility**				
Methicillin-resistant	38 (15·6)	48 (10·5)	62 (12·0)	0·14
**Severity**				
Severe Sepsis or Septic Shock on Day 0	99 (40·7)	135 (29·5)	264 (51·2)	<0·001
Hypotension (arterial blood pressure<70mmHG)	61 (25·1)	75 (16·4)	185 (35·9)	<0·001
Inotropes use	38 (15·6)	41 (9·0)	135 (26·2)	<0·001
Lowest Glasgow Score, median (IQR)	15 (14–15)	15 (14–15)	15 (12–15)	<0·001
**Laboratory analysis**				
Lowest O2 saturation in %, median (IQR)	94· (93–94)	95 (94–95)	94 (91–95)	<0·001
Highest FiO2 %, median (IQR)	25 (22–39)	27 (26–36)	29 (29–36)	<0·001
Serum CRP closest to first positive blood culture (mg/L), median (IQR)	185·0 (94·0–257·0)	78·0 (31·0–165·0)	172·0 (74·7–274·0)	<0·001
Serum Creatinine (mg/dL), median (IQR)	1·07 (0·79–1·74)	1·06 (0·73–2·02)	1·15 (0·75–2·05)	0·58
Neutrophil Count (x10^3/µL), median (IQR)	10·5 (7·0–14·0)	9·0 (6·5–12·0)	10·8 (7·5–13·5)	<0·001
Leukocytes Count (x10^3//µL), median (IQR)	12·7 (9·1–17·6)	10·0 (6·0–14·2)	12·0 (7·9–17·0)	<0·001

[1] Retrospectively judgement, more than one allowed

Table S9. Features and outcomes of patients in the FEN-AUREUS external validation cohort and in the phenotypes obtained.

Factor	A phenotype (n=283)	B phenotype (n=573)	C phenotype (n=329)	P value
**Demographics**				
Female sex	99 (35·0)	184 (32·1)	100 (30·4)	0·52
Median age in years (IQR)	66 (56–77)	66 (55–76)	71 (57–79)	0·001
**Comorbidities**				
Myocardial Infarction	39 (13·8)	130 (22·7)	58 (17·6)	0·006
Peripheral vascular disease	44 (15·5)	55 (9·6)	26 (7·9)	0·004
Dementia	21 (7·4)	17 (3·0)	40 (12·2)	<0·001
Chronic Lung Disease	27 (9·5)	66 (11·5)	51 (15·5)	0·05
Tumour	28 (9·9)	114 (19·9)	72 (21·9)	<0·001
Chronic renal disease	62 (21·9)	153 (26·7)	70 (21·3)	0·10
Chronic liver disease				0·03
No liver disease	246 (86·9)	483 (84·3)	296 (90·0)	
Mild	24 (8·5)	41 (7·2)	13 (4·0)	
Moderate or severe	13 (4·6)	49 (8·6)	20 (6·1)	
Diabetes with organ damage	60 (21·2)	73 (12·7)	25 (7·6)	<0·001
Diabetes without organ damage	63 (22·3)	123 (21·5)	82 (24·9)	0·48
Immunosuppressive Treatment	32 (11·3)	58 (10·2)	28 (8·5)	0·32
HIV Infection/Aids	3 (1·1)	3 (0·5)	2 (0·6)	0·95
Neutropenia on Day 0	6 (2·1)	14 (2·4)	11 (3·3)	0·70
Charlson Comorbidity Index (IQR)	2·00 (1·00–4·00)	2·00 (1·00–4·00)	2·00 (1·00–4·00)	0·09
**Acquisition of infection**				
Community Acquired	127 (44·9)	30 (5·2)	161 (48·9)	<0·001
Healthcare Associated	107 (37·8)	175 (30·5)	86 (26·2)	
Nosocomial	49 (17·3)	368 (64·3)	82 (24·9)	
**Main site of infection** [1]				
Skin and soft tissues excluding surgical wound and deep tissue	141 (49·8)	10 (1·7)	8 (2·4)	<0·001
Surgical wound	44 (15·5)	5 (0·9)	0 (0·0)	<0·001
Central venous catheter (including PICC)	8 (2·8)	216 (37·7)	0 (0·0)	<0·001
Peripheral venous catheter	2 (0·7)	262 (45·7)	0 (0·0)	<0·001
Infected intravascular thrombus	1 (0·4)	27 (4·7)	2 (0·6)	<0·001
Implanted vascular device	6 (2·1)	29 (5·1)	3 (0·9)	0·001
Native heart valve	17 (6·0)	7 (1·2)	8 (2·4)	0·005
Prosthetic heart valve	2 (0·7)	0 (0·0)	10 (3·0)	0·003

Epidural or intraspinal empyema	4 (1·4)	8 (1·4)	0 (0·0)	0·02
Vertebral bone/disc	18 (6·4)	14 (2·4)	4 (1·2)	<0·001
Native joint	21 (7·4)	21 (3·7)	3 (0·9)	<0·001
Prosthetic joint	2 (0·7)	13 (2·3)	2 (0·6)	<0·001
Other bone-related source	2 (0·7)	10 (1·7)	0 (0·0)	0·04
Deep tissue infection or abscess	17 (6·0)	5 (0·9)	17 (5·2)	<0·001
Pneumonia	8 (2·8)	10 (1·7)	103 (31·3)	<0·001
Source not established	10 (3·5)	9 (1·6)	79 (24·0)	<0·001
Other sources	11 (3·9)	5 (0·9)	39 (11·9)	<0·001
**Medical history**				
Previous non-bacteraemic *S. aureus* infection in the last 12 weeks	5 (1·8)	4 (0·7)	6 (1·8)	0·21
**Antibiotic susceptibility**				
Methicillin-resistant (MRSA)	42 (14·8)	43 (7·5)	32 (9·7)	0·003
**Physical examination data**				
Hypotension (arterial blood pressure<70mmHG)	54 (19·1)	106 (18·7)	79 (24·0)	0·12
Lowest Glasgow score,median (IQR)	15·00 (15·00–15·00)	15·00 (15·00–15·00)	15·00 (14·00–15·00)	<0·001
**Laboratory analysis**				
Lowest O2 saturation in %, median (IQR)	95 (95–97)	95 (95–96)	95 (94–97)	0·01
Highest FiO2 %, median (IQR)	21 (21–36)	21 (21–32)	21 (21–36)	0·19
Serum bilirubin (mg/dL), median (IQR)	0·63 (0·42–1·02)	0·69 (0·42–1·10)	0·66 (0·40–1·13)	0·60
Serum creatinine (mg/dL), median (IQR)	1·05 (0·74–1·99)	1·35 (0·77–2·15)	1·22 (0·80–1·95)	0·05
Serum CRP (mg/L), median (IQR)	185·8 (88·7–278·9)	110·3 (43·5–211·8)	180·8 (75·2–286·6)	<0·001
Neutrophil count (x10^3/µL), median (IQR)	10·4 (7·1–14·9)	8·1 (5·2–11·2)	10·3 (7·1–13·9)	<0·001
Lymphocyte count (x10^3/µL), median (IQR)	1·10 (0·60–1·60)	0·80 (0·40–1·20)	0·90 (0·50–1·30)	<0·001
Platelet count (x10^3/µL), median (IQR)	232 (154–342)	185 (127–272)	207(130–295)	<0·001

Figure S2. Kaplan-Meier survival analysis: 30-day mortality by phenotype and sub-phenotype in the INSTINCT external validation cohort

A) Kaplan-Meier survival analysis: 30-day mortality by phenotype in the INSTINCT external validation cohort

B) Kaplan-Meier Survival Analysis: 30-Day mortality by sub-phenotype in patients belonging to the A phenotype in the INSTINCT external validation cohort

C) Kaplan-Meier Survival Analysis: 30-Day mortality by sub-phenotype in patients belonging to the B phenotype in the INSTINCT external validation cohort

D) Kaplan-Meier Survival Analysis: 30-Day mortality by sub-phenotype in patients belonging to the C phenotype in the INSTINCT external validation cohort

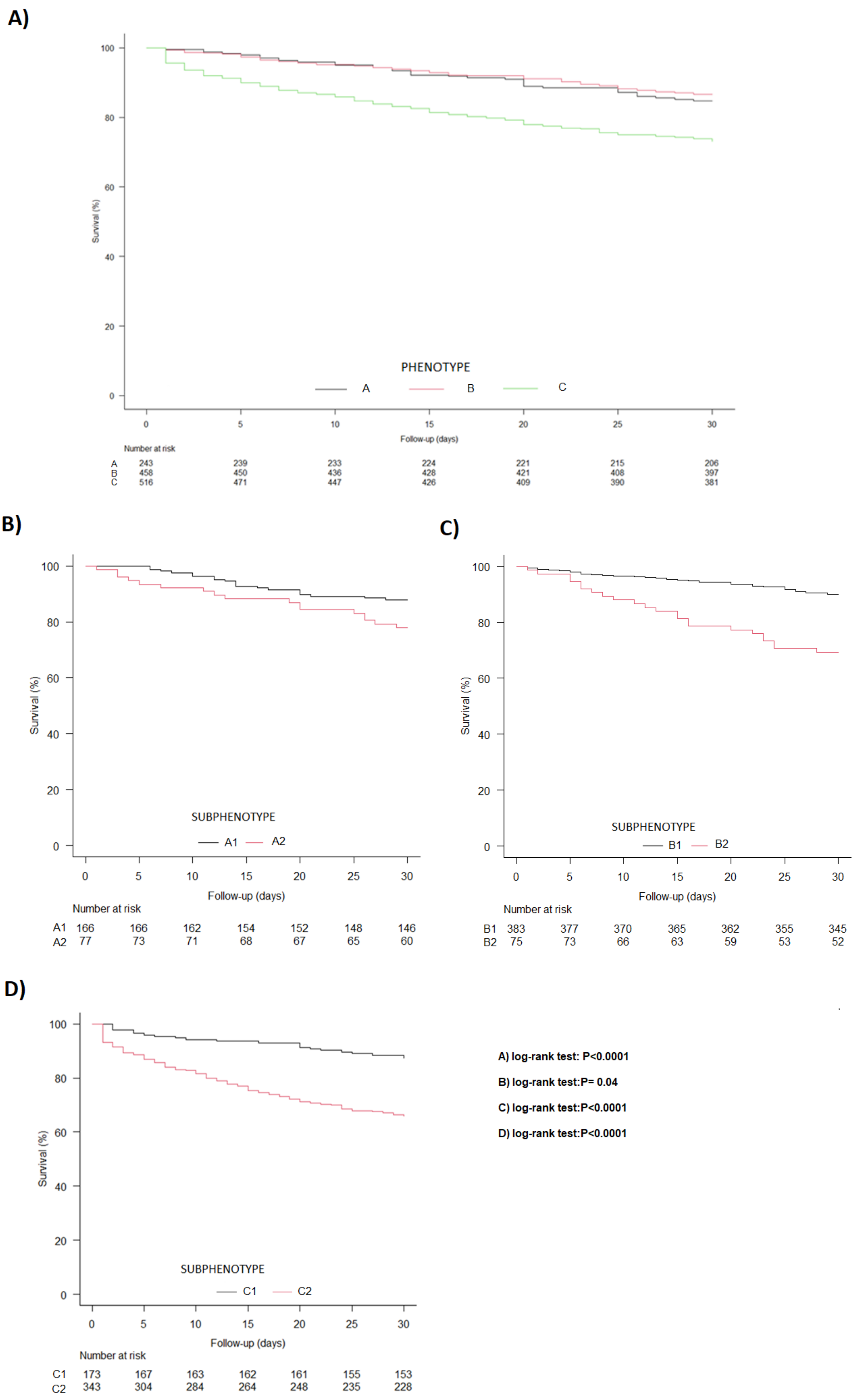

A) log-rank test: P<0.0001

B) log-rank test:P= 0.04

C) log-rank test:P<0.0001

D) log-rank test:P<0.0001

Table S10. Multivariate Cox regression of 30-day mortality for each of the identified phenotypes in the INSTINCT cohort[1]

VARIABLE	A		B		C	
	aHR (95%CI)	p	aHR (95%CI)	p	aHR (95%CI)	p
Source control within 3 days	0·98 (0·50–2·00)	0·95	0·63 (0·37–1·10)	0·10	0·87 (0·60–1·27)	0·47
Sub-phenotype 2	1·93 (1·01–3·71)	0·04	3·40 (2·02–5·72)	<0·0001	3·04 (1·84–5·02)	<0·0001

[1] Sub-phenotype 2 within each phenotype, based on the probable portal of entry, was identified using the single predictive model assigned to each phenotype according to the probable portal of entry.

Figure S3. Kaplan-Meier survival analysis: 30-day mortality by phenotype and sub-phenotype in the FEN-AUREUS external validation cohort

A) Kaplan-Meier survival analysis: 30-day mortality by phenotype in the FEN-AUREUS external validation cohort

B) Kaplan-Meier Survival Analysis: 30-Day mortality by sub-phenotype in patients belonging to the A phenotype in the FEN-AUREUS external validation cohort

C) Kaplan-Meier Survival Analysis: 30-Day mortality by sub-phenotype in patients belonging to the B phenotype in the FEN-AUREUS external validation cohort

D) Kaplan-Meier Survival Analysis: 30-Day mortality by sub-phenotype in patients belonging to the C phenotype in the FEN-AUREUS external validation cohort

A)

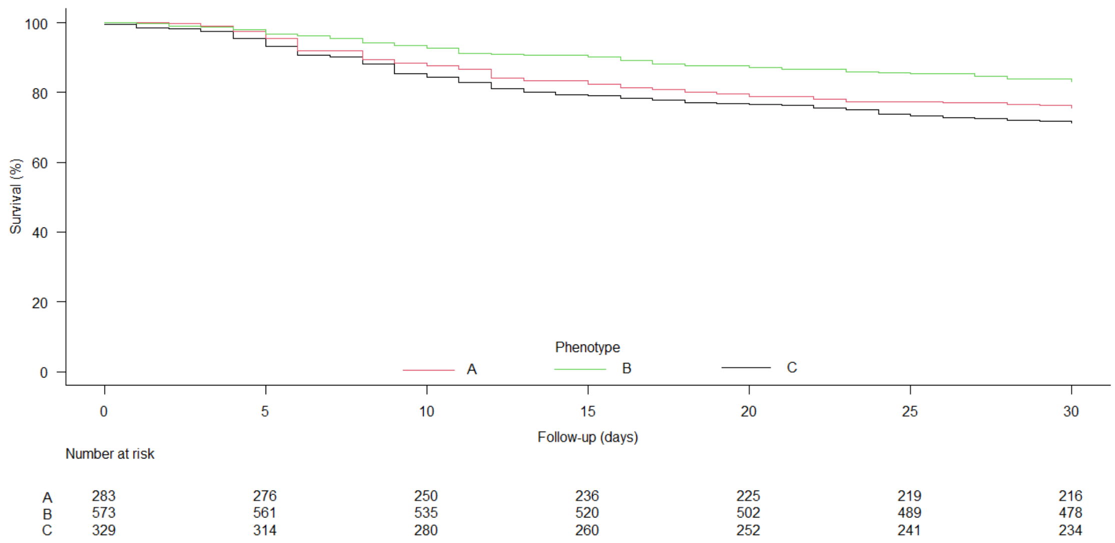
Survival (%)
100
80
60
40
20
0
Phenotype
A
B
C
0
5
10
15
20
25
30
Follow-up (days)
Number at risk
A 283 276 250 236 225 219 216
B 573 561 535 520 502 489 478
C 329 314 280 260 252 241 234

B)

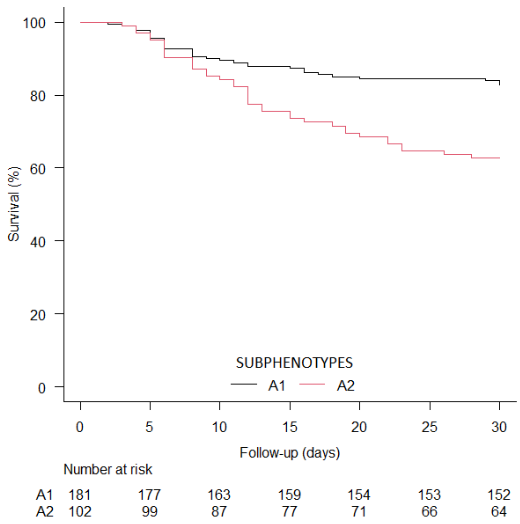
Survival (%)
100
80
60
40
20
0
SUBPHENOTYPES
A1
A2
0
5
10
15
20
25
30
Follow-up (days)
Number at risk
A1 181 177 163 159 154 153 152
A2 102 99 87 77 71 66 64

C)

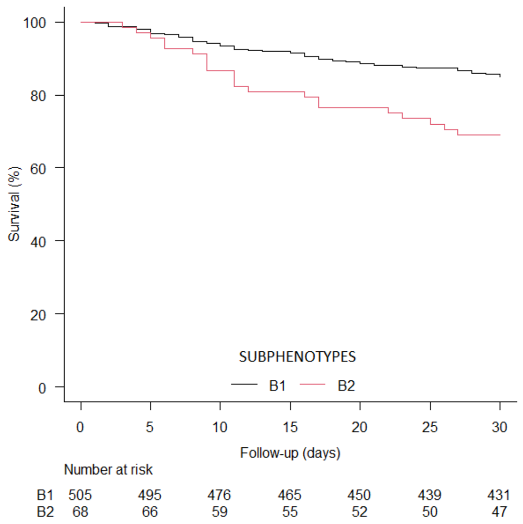
Survival (%)
100
80
60
40
20
0
SUBPHENOTYPES
B1
B2
0
5
10
15
20
25
30
Follow-up (days)
Number at risk
B1 505 495 476 465 450 439 431
B2 68 66 59 55 52 50 47

D)

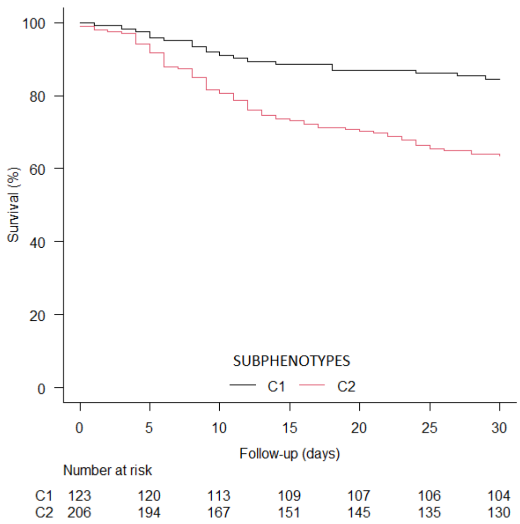
Survival (%)
100
80
60
40
20
0
SUBPHENOTYPES
C1
C2
0
5
10
15
20
25
30
Follow-up (days)
Number at risk
C1 123 120 113 109 107 106 104
C2 206 194 167 151 145 135 130

Table S11. Multivariate Cox regression of 30-day mortality for each of the identified phenotypes in the FEN-AUREUS cohort[1]

VARIABLE	A		B		C	
	aHR (95%CI)	p	aHR (95%CI)	p	aHR (95%CI)	p
Source control within 3 days	0·46 (0·20–1·08)	0·07	0·57 (0·35–0·93)	0·02	0·41 (0·13–1·32)	0·13
Sub-phenotype 2	2·02 (1·25–3·29)	0·004	2·11 (1·30–3·44)	0·002	2·44 (1·47–4·06)	0·0006
ID team visit	0·43 (0·25–0·74)	0·002	0·66 (0·40–1·10)	0·10	0·59 (0·38–0·90)	0·02

[1] Sub-phenotype 2 within each phenotype, based on the probable portal of entry, was identified using the single predictive model assigned to each phenotype according to the probable portal of entry.

OBSERVATIONAL POST-AUTHORIZATION STUDY WITH PROSPECTIVE FOLLOW-UP PROTOCOL

TITLE: Identification and validation of clinical phenotypes in *Staphylococcus aureus* bacteremia and their association with development of complicated bacteremia and mortality (FEN-AUREUS study)
PROTOCOL CODE: FIS-FENAUREUS-2022-01 TITLE:

EUDRACT no.: Not applicable

VERSION: 1.0 (March 2022)

COORDINATING INVESTIGATOR: Belén Gutiérrez Gutiérrez

SPONSOR: Fundación Pública Andaluza para la gestión de la Investigación en Salud de Sevilla (FISEVI)
[Andalusian Public Foundation for the Health Research Management in Seville]
Avda. Manuel Siurot, s/n – Edificio de Laboratorios, 6ª planta – 41013 Seville

The information contained in this document is confidential and the property of the Fundación Pública para la Gestión de la Investigación en Salud de Sevilla (FISEVI) and may not be disclosed to others without the written permission of the investigators, except for the use that may be made of it to obtain informed consent from those persons who will receive the investigational medicinal product, and in communications with health authorities, clinical trial committees, or those persons who will conduct the study.

La información contenida en este documento es confidencial y es propiedad de la Fundación Pública Andaluza para la Gestión de la Investigación en Salud de Sevilla y no podrá ser revelada a otras personas sin autorización por escrito de los investigadores, a excepción del uso que de ella pueda hacerse para obtener el consentimiento informado de las personas que vayan a recibir el fármaco en investigación, así como en comunicaciones a las autoridades sanitarias, los comités de ensayos clínicos o aquellas personas que vayan a llevar a cabo el estudio.

SIGNATURE OF THE SPONSOR

I have read the protocol entitled "Identificación y validación de fenotipos clínicos en bacteriemias por *Staphylococcus aureus* y su asociación con la mortalidad y desarrollo de bacteriemia complicada (Identification and validation of clinical phenotypes in *Staphylococcus aureus* and their association with the development of complicated bacteremia and mortality) (FEN-AUREUS study)", version 1.0, dated March 7, 2022 and agree to abide by all its terms and to comply with all applicable laws and regulations, including, but not limited to the International Conference on Harmonization (ICH) Good Clinical Practice (GCP) guidelines and the ethical principles of the Declaration of Helsinki

José Cañón Campos
On behalf of the Fundación Pública Andaluza para
La Gestión de la Investigación en Salud de Sevilla (FISEVI)
Position: Managing Director

Signature:
Date:

FIRMA DEL INVESTIGADOR COORDINADOR

I have read the protocol entitled "Identificación y validación de fenotipos clínicos en bacteriemias por *Staphylococcus aureus* y su asociación con la mortalidad y desarrollo de bacteriemia complicada (Identification and validation of clinical phenotypes in *Staphylococcus aureus* and their association with the development of complicated bacteremia and mortality) (FEN-AUREUS study)", 1.0, dated March 7, 2022 and agree to abide by all its terms and to comply with all applicable laws and regulations, including, but not limited to the International Conference on Harmonization (ICH) Good Clinical Practice (GCP) guidelines and the ethical principles of the Declaration of Helsinki

Dr. Belén Gutiérrez Gutiérrez
Coordinating Investigator

Firma:
Fecha:

SIGNATURE OF THE PRINCIPAL INVESTIGATOR OF THE CENTER

I have read the protocol entitled "Identificación y validación de fenotipos clínicos en bacteriemias por *Staphylococcus aureus* y su asociación con la mortalidad y desarrollo de bacteriemia complicada (Identification and validation of clinical phenotypes in *Staphylococcus aureus* and their association with the development of complicated bacteremia and mortality) (FEN-AUREUS study)", version 1.0, dated March 7, 2022 and agree to abide by all its terms and to comply with all applicable laws and regulations, including, but not limited to the International Conference on Harmonization (ICH) Good Clinical Practice (GCP) guidelines and the ethical principles of the Declaration of Helsinki

Dr.

Principal Investigator Signature: ____________________

Center: Hospital……. Date: ____________________

Index

ABBREVIATIONS AND DEFINITIONS.. 6
RESPONSIBLE FOR THE STUDY... 7
PROTOCOL SUMMARY.. 8
WORK PLAN, FOLLOW-UP .... …………10
GENERAL AND SPECIFIC OBJECTIVES: PILLARS……………………………. 11
CRITICAL REVIEW OF THE LITERATURE... 12
METHODS... 13
ETHICAL CONSIDERATIONS /PROTECTION OF PARTICIPATING SUBJECTS ……............................16
MANAGEMENT AND REPORTING OF ADVERSE REACTIONS……. 17
PLANS FOR THE DISSEMINATION OF RESULTS. 18
RESOURCES FOR THE CONDUCT OF THE STUDY AND ASSIGNMENT OF TASKS; FUNDING.........19
BIBLIOGRAPHY.. 20
PROTOCOL MODIFICATIONS………... .22
PRACTICALCONSIDERATIONS:….. 23
ANNEXES.. 24
Annex 1: Data collection notebook ……….. 24
Annex 2: Commitment of the coordinating investigator.. 24
Annex 3: Compliance of the CEIC. 24
Annex 4: Subject information sheets…… 24
Annex 6: Informed consent form……………………... 24
Annex 7: Financial report ……... …… 24

ABBREVIATIONS AND DEFINITIONS

MIC: Minimum inhibitory concentration

S. aureus: Staphylococcus aureus

IV: intravenous;

HU: University Hopsital

HUVM: University Hospital Virgen Macarena

HUVV: University Hospital Virgen de Valme

Community presentation: Infection occurring in a patient who was not hospitalized during the previous week, or starts within 72 hours of a hospital admission (this definition includes community infections and non-nosocomial healthcare-related infections).

Adverse Event: Any adverse of unfavorable event that appears in a study participant, including any sign, symptom or disease, whether or not considered to be related to the drug administered.

SPONSOR

FUNDACIÓN PÚBLICA ANDALUZA PARA LA GESTIÓN DE LA INVESTIGACIÓN EN SALUD DE SEVILLA (FISEVI)

(Andalusian Public Foundation for the Health Research Management in Seville)

PROTOCOL SUMMARY:

1. Sponsor identification and address.

FUNDACIÓN PÚBLICA ANDALUZA PARA LA GESTIÓN DE LA INVESTIGACIÓN EN SALUD DE SEVILLA (FISEVI)

H.U. Virgen del Rocío

Avda. Manuel Siurot, s/n

Edificio de Laboratorios, 6ª planta

41013 Sevilla

Tel (Macarena site): 600 162 458

Fax (Macarena site): 95 500 80 15

2. Title of the study.

"Identificación y validación de fenotipos clínicos en bacteriemias por *Staphylococcus aureus* y su asociación con la mortalidad y desarrollo de bacteriemia complicada (FEN-AUREUS study)" (Identification and validation of clinical phenotypes in *Staphylococcus aureus* bacteremia and their association with development of complicated bacteremia and mortality).

3. Protocol code (according to official coding guidelines).

FIS-FENAUREUS-2022-01

4. Principal Investigators and center.

INVESTIGADORES PRINCIPALES	CENTRO
Belén Gutiérrez-Gutiérrez	Hospital Universitario Virgen Macarena (Sevilla) España
Marta Trigo	Hospital Universitario Virgen de Valme (Sevilla) España
Walter Alfredo Goycochea	Hospital Universitario Virgen del Rocío (Sevilla) España
Francisco Javier Martínez Marcos	Hospital Universitario Juan Ramón Jiménez (Huelva) España
Ana Arizcorreta Yarza	Hospital Universitario Puerta del Mar (Cádiz) España
María Luisa Fernández Ávila	Hospital Universitario de Puerto real (Cádiz) España
Salvador López Cárdenas	Hospital Universitario de Jerez de la Frontera (Cádiz) España
Antonio Plata Ciézar	Hospital Universitario Regional de Málaga (Málaga) España
Rosario Palacios Muñoz	Hospital Universitario Virgen de la Victoria (Málaga) España
Angela Cano	Hospital Universitario Reina Sofía (Córdoba) España
Miguel Ángel López	Hospital Universitario Virgen de las Nieves (Granada) España
Francisco Anguita Santos	Hospital Universitario de San Cecilio (Granada) España
Gaspar Duro Ruiz	Complejo hospitalario Universitario de Jaén (Jaén) España
Alexandra Aceituno Caño	Hospital Universitario Torrecárdenas (Almería) España
Ana Belén Lozano Serrano	Hospital Universitario Poniente (Almería) España
Marco Piscaglia	Luigi Sacco Hospital (Milan) Italia
Alessandra Oliva	Azienda Ospedaliero Universitaria Policlinico Umberto I, Department of Public Health and Infectious Diseases, Sapienza University of Rome (Rome), Italia

5. Types of center where the study is planned to be carried out.

The study involves 17 centers in Andalusia HUVM (Coordinating center), HUVV and HU Virgen del Rocío (Sevilla); HU Reina Sofía (Córdoba), HU Puerta del Mar, HU de Puerto Real and H de Jerez (Cádiz); H. Juan Ramón Jímenez (Huelva); HU Virgen de la Victoria and HU Regional de Málaga (Málaga); HU Virgen de las Nieves and HU San Cecilio (Granada); H. Universitario Torrecárdenas (Almería) and Complejo Hospitalario Universitario de Jaén (Jaén).

6. Research Ethics Committee (REC) evaluation

Requires the favorable opinion of the Andalusian Biomedical Research Ethics Coordinating Committee (in process).

7. Primary objective.

To determine retrospectively whether different clinical phenotypes exist among patients (adults and children) who develop *Staphylococcus aureus* bacteremia.

Secondary objectives:

Secondary objectives:

1 To evaluate the reproducibility of these clinical phenotypes and their correlation with mortality (30 days and 6 months)

2 To derive and validate a simplified probabilistic model of phenotype assignment.

3 External validation of the simplified probabilistic model of clinical phenotype assignment in a prospective cohort, and determination of associations with mortality and the development of complicated bacteremia within 6 months.

4 To apply microbiological (clonality testing, genome sequencing), biochemical and immunological techniques to elucidate the pathophysiological and genetic mechanisms underlying the phenotypes.

8. Design.

This is an entirely observational study.

Primary Objective and Secondary Objectives 1 and 2: The ISAC cohort will be analyzed. This cohort is the result of an international study (11 hospitals in 5 countries) on bacteremia caused by *S.aureus* (2,590 cases collected) in which the University Hospital Virgen Macarena participated. The other participating centers have granted authorization to carry out the analysis for the FEN-FENAUREUS project.

Secondary Objectives 3 and 4: This will be a multicenter prospective cohort study. Each patient who develops *S. aureus* bacteremia will be assigned a phenotype and will be followed for 6 months to evaluate differences in mortality and development of complicated bacteremia according to phenotype.

For Secondary Objective 5: This study of genetic (microorganism and the patient), immunological, biochemical and microbiological variables will be performed on representative patients, selected from the prospective cohort (two centers HUVM and HUVV), with the different phenotypes obtained.

Inclusion criteria: adults and children with clinically significant *S. aureus* bacteremia (i.e., associated with at least 2 of the classic criteria for systemic inflammatory response: fever, tachycardia, tachypnea, decreased level of consciousness, hypotension, leukocytosis/leukopenia, or organ failure).

For the study of genetic, immunological, biochemical and microbiological variables in representative patients, a predefined number of patients with each of the phenotypes assigned by the probabilistic model will be randomly selected from the prospective cohort (at the HUVM and HUVV).

Exclusion criteria: Non-clinically significant bacteremia; lack of follow-up blood cultures at 48-72 hours; patients who died in the first 48 hours; subsequent episodes in the same patient.

9. Disease or disorder under study.

Clinically significant *S. aureus* bacteremia

10. Details of the drugs under study.

The type of antibiotic treatment administered will be only one of the explanatory variables that will be collected (indicated below) and recorded in order to achieve the proposed objectives set out above. Therefore this study IS NOT CONSIDERED AN OBSERVATIONAL STUDY WITH MEDICINES, AS IT DOES NOT FALL UNDER ANY OF THE SUPPOSITIONS GIVEN IN ARTICLE 2 OF ROYAL DECREE 957/2020, OF NOVEMBER 3, REGULATING OBSERVATIONAL STUDIES WITH MEDICINES FOR HUMAN USE, and in no case does it alter the prescribing practice of the physician, or the dispensing practice of the pharmacist. The prescription of medication will follow the usual pathway.

11. Study population and total number of subjects

For the retrospective study (objectives 1-3) based on the ISAC database, we estimate that we will be able to analyze at least 1,800 cases of SAB that meet the inclusion criteria. Likewise, for objective 4, we estimate that 1,000 cases will be prospectively included from the 14 participating centers during the 34 months of follow-up. For objective 5, given that we estimate that 3 or 4 phenotypes will be identified, 100 patients/ isolates will allow us to include 25-33 per phenotype, which will allow a reasonable number of cases for comparison of microbiological, genetic, endothelial and immunological parameters.

12. Schedule (quarterly periods indicated)

- January to May 2022: Retrospective cohort analysis of ISAC.

- March to May 2022: Registration in the REec (Spanish Registry of Clinical Trials) (if considered necessary); preparation of electronic datasheets; submission of documentation to the Andalusian Biomedical Research Ethics Coordinating Committee; sending of documentation to participating centers; piloting of the data collection sheet.
- June 2022 to June 2024: Inclusion of patients in prospective cohort, sample collection; data collection and recording in electronic datasheet.
- September 2022 to June 2024: Study of genetic (microorganism and patient), immunological, biochemical and microbiological variables.
- June 2022 to June 2024: Data monitoring; database review for missing/inconsistent data. Closure of database.
- Third quarter 2024: Data analysis.
- Fourth quarter 2024: Publication of results.

13. Source of funding.

There is no financial compensation for patients or investigators, nor are there any undeclared sources of funding that could lead to conflicts of interest in the research results.

WORK PLAN (TASKS, MILESTONES AND STUDY TIMELINE.

**Month-Year**	**Task**	**Responsible**
January 2022-May 2022	Retrospective cohort analysis of ISAC	Principal investigators
March 2022-May 2022	Registration in REec; preparation of electronic datasheet; submission of documentation to the Andalusian Biomedical Research Ethics Coordinating Committee; sending of documentation to participating centers; piloting of data collection sheet	Principal investigators
June 2022-June 2024	Inclusion of patients in prospective cohort, sample collection; data collection and recording in electronic datasheet	Principal investigators and collaborators
September 2022 to June 2024	Study of genetic (microorganism and patient), immunological, biochamical and microbiological variables.	
June 2022 to June 2024	Data monitoring; database review for missing/inconsistent data.	Principal investigators and

	Closure of database.	collaborators
July 2024- September 2024	Analysis of results	Principal investigators and collaborators
October 2024-December 2024	Publication of results	Principal investigators and collaborators

GENERAL AND SPECIFIC OBJECTIVES: BASES

Primary Objective

To determine retrospectively whether patients (adults and children) who developed bacteremia caused by *Staphyclococcus aureus* had different phenotypes.

Secondary Objectives

1 To evaluate the reproducibility of the clinical phenotypes and their correlation with mortality (30 days and 6 months).
2 To derive and validate a simplified probabilistic model of clinical phenotype assignment.
3 External validation of the simplified probabilistic model of clinical phenotype assignment in a prospective cohort and determination of associations with mortality and the development of complicated bacteremia within 6 months.
4 To apply microbiological (clonality testing, genome sequencing), biochemical and immunological techniques to elucidate the pathophysiological and genetic mechanisms underlying the phenotypes.

CRITICAL REVIEW OF THE LITERATURE.

Staphylococcus aureus is one of the main microorganisms that cause disease in humans and one of the main causes of bloodstream infections (bacteremia) worldwide. *S. aureus* bacteremia causes significant morbidity and increases healthcare costs; complications are frequent and mortality ranges from 20-40%. As a result, the concept of "complicated bacteremia" has been introduced. Predictors of complicated bacteremia are the persistence of bacteremia after 2-3 days of active treatment, the appearance of secondary foci of infection or infection of prosthetic valves and other prosthetic implants, endocarditis and specific cutaneous/mucosal lesions. More specifically, persistent bacteremia is considered a good predictor of possible complications and is a criterion for prolongation of antimicrobial treatment for 4 weeks. At the present time, the identification of

patients who develop complicated bacteremia is performed during the initial follow-up of the patient, pending the results of control blood cultures and other indicated diagnostic tests such as echocardiography. However, early identification of patients at high risk of developing complicated bacteremia would be of great use in planning treatment, monitoring for complications and evaluating more aggressive therapeutic strategies in this subgroup of patients. Independently of this, the pathogenesis of persistent bacteremia, when not due to endocarditis or an undrained focus, is not well established.

The approach outlined in this research project proposal is based on the hypothesis that patients who develop *S. aureus* bacteremia could be categorized into a few clinical patterns (phenotypes) based on demographic characteristics, comorbidities, history, signs, symptoms, and laboratory tests at presentation. Such phenotypes, if they exist, could be indicative of different physiopathological mechanisms, as well as be associated with a better or worse prognosis. They could help to better identify patients with *S. aureus* bacteremia at higher risk of complications and those who are not, which could improve treatment strategies and patient follow-up, resulting in a reduction in mortality and complications associated with this type of infection. In addition, the analysis of possible genetic, immunological and pathophysiological mechanisms that would explain the clinical presentation of bacteremia according to the phenotypes identified would lead to the investigation of new treatment strategies. Consequently, our proposal for this Research Project also includes a study of phenotypes associated with a higher or lower risk of developing complications and mortality, and an analysis of their differences based on genetics, microbiology (presence of virulence genes), immunological and physiopathological factors such as endothelial dysfunction (early biomarkers of endothelial dysfunction) and cytokine dysregulation.

Given the particular characteristics of the pediatric population, a specific subanalysis of this population is also foreseen. The Andalusian pediatric infectology and immunopathology group will contribute to the project for this part of the project.

Presentation according to different clinical phenotypes has been described in different pathologies but never in *S. aureus* bacteremia. For example, one study identified clinical phenotypes among patients with sepsis (Seymour et al. JAMA 2019). Our group also recently identified three clinical phenotypes associated with prognosis in patients with COVID-19 requiring hospital admission (Gutierrez-Gutierrez et al, Lancet Infect Dis 2021). It is considered that this approach to the study of such a serious pathology could be of great utility for the clinical management of patients (both in the adult and pediatric populations) and its results of great interest to the medical and scientific community.

METHODS:

Design and justification.

This is an entirely observational study.

For the Primary Objective and Secondary Objectives 1 and 2: the ISAC cohort will be analyzed. This cohort is the result of an international study (11 hospitals in 5 countries) on bacteremia caused by *S. aureus* in whicih the Hospital Virgen Macarena participated (2,590 cases have been collected, and it is estimated that at least 1,800 meet the criteria for inclusion). Authorization to perform the analysis for the FEN-FENAUREUS project has been obtained from the rest of the participating centers.

For Secondary Objectives 3 and 4 a multicenter prospective cohort study will be performed. All patients who develop *S. aureus* bacteremia will be assigned a phenotype and followed for 6 months to evaluate differences in mortality and development of complicated bacteremia between phenotypes.

For Secondary Objective 5 a study of genetic (microorganism and patient), immunological, biochemical and microbiological variables will be performed in representative patients with different phenoytpes; these patients will be selected from the prospective cohort at two centers (HUVM and HUVV).

2. Study population.

ISAC and prospective cohorts: Criteria for inclusion: adults and children (prospective cohort only) with clinically significant *S. aureus* bacteremia (i.e., associated with at least 2 classic criteria for systemic inflammatory response: fever, tachycardia, tachypnea, decreased level of consciousness, hypotension, leukocytosis/leukopenia, or organ failure).

For the study of genetic, immunological, biochemical and microbiological variables in representative patients: a predetermined number of patients belonging to each phenotype assigned by the probabilistic model will be randomly selected from the prospective cohort (HUVM and HUVV centers).

Exclusion criteria: non-clinically significant bacteremia; lack of follow-up blood cultures at 48-72 hours; patients who died in the first 48 hours; subsequent episodes in the same patient.

3. Source of information.

The source of information for the retrospective cohort will be the ISAC database. For the prospective cohort, the clinical history collected during follow-up visits of the patients in that cohort will be used.

4. Operational definitions of outcome, exposure and other variables.

- Outcome variables:
  - Primary outcome variable: all-cause mortality at day 30;

Secondary outcome variable: all-cause mortality at month 6 (measured as time-to-death, prospective cohort only). Persistent bacteremia (positive blood culture 2-3 days after initiation of appropriate targeted therapy). Complicated bacteremia (considered as presence of endocarditis, secondary foci, recurrence within 6 months). Hospital stay.

-
-

Explanatory: age, sex, service, type of acquisition (community-acquired, healthcare-related, nosocomial [Friedman criteria]; types of relationship with previous care), underlying diseases (diabetes, chronic lung disease, chronic renal insufficiency, chronic liver disease, immunosuppressive disorders, cancer, other), severity of underlying disease (Charlson index, McCabe classification), Barthel index, neutropenia, parenteral drug use, immunosuppressive treatment, recent invasive procedures (dialysis, catheter and vascular type, urinary catheter, mechanical ventilation, endoscopy, vascular procedures, surgery, percutaneous puncture), antibiotic therapy in the previous month, antibiotic susceptibility of *S. aureus*, Pitt and SOFA score on day 0 (blood cultures performed), sepsis or septic shock (Singer et al. JAMA 2016 for adults and Dellinger et al, Intensive Care Medicine 2013//Mathias et al, Curr Opin Pediatr 2016 for pediatric patients), acute kidney injury, empiric and targeted antibiotic therapy (drug, dose, route, day of onset and termination, reason for termination), source of bacteremia (clinical and microbiological criteria), management of source of infection (removal of catheter or other device, drainage), supportive care, echocardiographic data, if performed (transthoracic/transesophageal), CBC and basic blood chemistry at 0.

Exposure variables**:** Type of phenotype

-

5. Expected sample size and basis for determination.

For the retrospective study (objectives 1-3) based on the ISAC database: we estimate that we will be able to analyze at least 1,800 cases of SAB that meet the inclusion criteria. For objective 4: we estimate prospective inclusion of 1000 cases from the 14 participating centers during 34 months of follow-up.

For objective 5: we estimate that 3 or 4 phenotypes will be identified, and therefore that 100 patients/isolate will be sufficient to provide 25-33 patients per phenotype, which will allow a reasonable number of cases for comparison of microbiological, genetic, endothelial and immunological parameters.

6. Data collection methods (prospective cohort).

Data will be collected by prospectively obtaining information from DIRAYA of SAB episodes detected by the microbiology service at each of the participating Andalusian centers. Access to the electronic database, as well as prospective data collection to complete the electronic database, will be direct, remote, and carried out centrally by the HUVM investigation team in conjunction with the local teams at each center. Patients will also be followed up for 6 months afterwards by center investigators.

Data will be collected in an eDCN (electronic data collection notebook) developed for the purpose:

eDCN

A specific eDCN will be developed for this study. Based on experience with previous projects in SAB and using a tool developed by REIPI investigators that has already been registered, validated, and successfully tested in previous studies such as the FOREST, SIMPLIFY and PROBAC studies, a DCN will be designed that is intuitive for the user and clear and easy to analyze. Access to the eDCN will likely be through staff passwords only. Secure systems are used to ensure confidentiality, integrity and security. All data in the eDCN will be anonymized.

Sampling

The study of genetic, immunological, biochemical and microbiological variables in representative patients with the different phenotypes will be performed only at the following centers: HUVM and HUVV. Samples obtained during routine patient care will be used and no additional samples will be taken. All participants in this substudy will be informed and specific written informed consent will be obtained.

Data sharing / Ethical issues

In accordance with current recommendations for data sharing, data from this study will be available to other investigators for meta-analysis following a written proposal, approval by the Ethics Committee and the PI of the study and the sponsor, and signing of an agreement with the PI and sponsor (FISEVI).

This is an observational study in which the main study variable is the identification of clinical phenotypes, with the application of microbiological (clonality testing, genome sequencing) biochemical and immunological techniques to elucidate the physiopathological and genetic mechanisms underlying the phenotypes. Hence, the study carries no risk for patients, since it does not involve any intervention or any additional or different treatment to that which patients would receive if they did not partipate in the study. As indicated above, it is not considered an observational study with medicines according to the stipulations of Royal Decree 957/2020,

which regulates observational studies with medicines for human use. There will be no financial benefit for patients if they participate in the study. The data relating to the prospective cohort will be collected in a research file (electronic DCN) for which the Fundación Pública Andaluza para la Gestión de la Investigación en Salud de Sevilla (FISEVI) is responsible and which will adopt the appropriate measures to ensure the protection of data privacy.

The protocol developed as a result of this Project proposal will be evaluated by the CEIm for Andalusia.

This protocol will comply with the principles of the Declaration of Helsinki for research projects involving human subjects.

All personal data necessary for the development of the study will be collected and processed in accordance with the provisions of Regulation (EU) 2016/679 of the European Parliament and of the Council of 27 April 2016 on the protection of natural persons with regard to the processing of personal data and on the free movement of such data, and repealing Directive 95/46/EC (General Data Protection Regulation), with current Spanish legislation on data protection, and Article 16. 3 of Law 41/2002, of November 14, 2002, which regulates patient autonomy and the rights and obligations regarding clinical information and documentation.

Microbiological studies:

At all participating centers: standard microbiological methods will be used, following SEIMC recommendations for the diagnosis of bacteremia (http://www.seimc.org/documentos/protocolos/microbiologia). For sensitivity testing, EUCAST guidelines will be followed.

Studies to meet Objective 5 (100 isolates selected at HUVM and HUVV): 1. Identification: Isolates will be identified by MALDI-TOF.

2. Sensitivity testing: The sensitivity of the 100 *S.aureus* isolates to the following antimicrobials will be tested by broth microdilution: penicillin, amoxicillin/clavulanic acid, oxacillin, gentamicin, tobramycin, amikacin, ciprofloxacin, levofloxacin, erythromycin, clindamycin, tetracycline, trimethoprim/sulfamethoxazole, linezolid, teicoplanin, vancomycin and daptomycin, following the EUCAST methodology.

3. Mass sequencing: Extraction of DNA from isolates will be performed using a Qiacube automatic extractor (Qiagen). The highly sensitive Qubit system (Thermo Fisher Scientific) will be used for fluorimetric quantification of extracted DNA. Enzymatic fragmentation of the DNA, addition of adapters and indexes, PCR amplification and subsequent clean-up, and library normalization will be performed according to the manufacturer's instructions (Nextera DNA Flex library prep kits [Illumina]. Library quality will be checked on the

Agilent 2100 Bioanalyzer system using a high-sensitivity chip. Library mixing and sequencing will be performed on a Miseq sequencer (Illumina), using the Illumina MiSeq v3 reagent kit (600 cycles and 2x300 paired-end reads). Reads will be quality-filtered and assembled with CLC Genomic Workbench v10 (Qiagen) software. The RAST server will be used for gene annotation. FastQ files generated for each sample will be used for genotyping and identifying genes of interest (ResFinder and VirulenceFinder of the Center for Genomic Epidemiology CGE], http://www.genomicepidemiology.org/). These databases will also be used to establish clonal relatedness between strains using multilocus sequence typing (MLST finder).

OTHER METHODS (100 patients sorted by different phenotypes, from HUVM and HUVV):

Study of dysregulated cytokine production: Blood samples from 100 patients will be tested to determine differences between the phenotypes for the main markers of cytokine dysregulation. Cytokine and adhesion molecule levels will be determined by Bio-Plex assay. EDTA tubes will be used for the collected samples. Blood samples will be centrifuged for 15 min at 1000 g, aliquoted into 0.2 mL tubes, and stored at -80ºC until analysis. For each sample, the Bio-Plex multiplex immunoassay will be used to simultaneously quantify (working in microplates) up to 12 human cytokines. The cytokines to be analyzed will be chosen from among the following: basic FGF, eotaxin, G-CSF, GM-CSF, IFN-γ, IL-1β, IL-1ra, IL-1α, IL-2Rα, IL-3, IL-12 (p40), IL-16, IL-2, IL-4, IL-5, IL-6, IL-7, IL-8, IL-9, GRO-α, HGF, IFN-α2, LIF, MCP-3, IL-10, IL-12 (p70), IL-13, IL-15, IL-17A, IP-10, MCP-1 (MCAF), MIG, β-NGF, SCF, SCGF-β, and SDF-1α.

Study of markers of endothelial dysfunction: Differences in the main markers of endothelial dysfunction (plasma levels of E-selectin, VCAM-1 and ICAM-1) in the 100 representative samples. For determination of adhesion molecule levels, EDTA and heparin plasma samples (for measurement of E-selectin) will be collected and centrifuged within 30 minutes of blood collection. Subsequent aliquots will be stored at -80ºC until analysis: E-selectin, VCAM-1 and ICAM-1 levels will be assessed with commercially available ELISA kits and a photometer (Tecan Sunrise, Craisheim, Germany) measured at 450 nm.

Immunological study: quantification of immunosuppression by flow cytometry: Whole blood drawn into K3-EDTA collection tubes will be used for flow cytometry analysis (BD Facs Canto II) of different subpopulations. For analysis of the main lymphocyte subpopulations (LT, LB, NK, CD4, CD8), a 6-color antibody panel (for CD45, CD3, CD4, CD8, CD16+56, CD19) will be used, and for Treg lymphocytes, a 3-color antibody panel (CD4, CD25 and CD127). Analyses of activated CD4 and CD8 lymphocytes: OX40+, HLA-DR+,CD28, inhibited lymphocytes (PD-1, CTLA-4), and myeloid-derived suppressor cells (MDSC) with simultaneous

labelling of CD45, CD33, CD11b, HLA-DR, CD14 and CD15. Dendritic cells: Lin- CD45 + CD11c- CD11b + CD123 + HLA-ABChigh HLA-DRhigh CCR7high OX40L + DC-SIGN + CD86highCD80highCD40highCD83+.

Genetic study of the patients: Whole blood samples will be taken from each patient. These samples will be immediately frozen at -80ºC and then sent to the Hospital Universitario de Valme where they will be cryopreserved awaiting genetic analysis. DNA will be isolated from frozen whole blood samples using the Qiacube system (Qiagen, Hilden, Germany) according to the manufacturer's instructions, and its concentration measured by fluorimetry, using the Qubit 3 fluorometer (Thermo Scientific, Wilmington, USA). Candidate gene variants identified in the literature will be analyzed using the AriaMX system (Agilent Technologies, Santa Clara, USA) and Taqman probes. Quality controls for genotyping and genetic association studies will be performed as previously described (Real LM, et al. A polymorphism linked to RRAS, SCAF1, IRF3 and BCL2L12 genes is associated with cirrhosis in hepatitis C virus carriers. Liver Int 2014; 34:558-66).

7. Data management.

Data will be collected in the electronic DCN online. Each participating center will be assigned a key and password to access the electronic DCN online and enter the data / variables required for each case. DCNs will be numbered with a code to ensure sample and data confidentiality.

8. Data analysis.

ISAC Cohort: Objectives 1 and 2: Missing value analysis and imputation, if applicable, will be performed using standard techniques. To derive the phenotypes, a two-step cluster analysis with silhouette analysis will be performed on a randomly selected subcohort including two-thirds of the patients. The phenotypes will be represented as chord diagrams and heat maps. The distribution of phenotypes and characteristics will be validated on the other subcohort with one-third of the patients. Multivariate analysis (Cox or logistic regression) will be performed to control for the effects of antibiotic treatment and control of focus of infection on the association between the identified phenotypes and the outcome variable. Objective 3: A binary (if two phenotypes are obtained) or multinomial (if >2) logistic regression model will be used to predict the phenotypes obtained. A simplified probabilistic model of phenotype assignment will then be developed based on the final logistic regression model selected.

Prospective cohort: (Objective 4). Each patient with SAB will be assigned a phenotype that will be calculated on the basis of the simplified probabilistic model. Mortality rates (at 30 days and six months), persistent bacteremia and complicated bacteremia will be compared between the different types of phenotypes. Multivariate analyses (Cox or logistic regression) will be calculated to control for the effect of other covariates

(such as treatment administered or source control) on the association between identified phenotypes and outcome variables.

Study of genetic, immunological, biochemical and microbiological variables in representative patients with different phenotypes, randomly selected from the prospective cohort of HUVM and HUVV cases (Objective 5): Differences in frequencies or medians of the data for genetic virulence factors, biomarkers or immunological factors across the different phenotypes will be analyzed by the chi-square or Mann-Whitney U test and multivariate logistic regression models, taking into account possible confounding variables.

CONSIDERATIONS IN PEDIATRIC PATIENTS

We estimate that 340 cases will be prospectively included from 17 participating centers during the 34-month follow-up.

Microbiology and immunology testing procedures will be similar to those in the adult cohort, adjusting blood collection volume to the maximum allowed for age and weight in pediatric patients.

Recent studies have suggested the involvement of an altered IL17 immune response in the context of invasive infections (CID 2019, PMID: 30165412), although it is most commonly described in the context of *S. aureus* of the skin and mucous membranes. Consequently, we propose to determine circulating IL-17A and Th17 (CD4+CXCR3-CCR6 +CCR4+ CD161+) levels at the time of blood culture sampling in the prospective cohort (Laboratorio 205, “Errores Innatos de Inmunidad”, Ibis; Laboratory 205, “Inborn Errors of Immunity”, Ibis).

9. Quality control.

All personal data necessary for the conduct of the study will be collected and processed in accordance with the provisions of Order SAS/3470/2009 on observational post-authorization studies for medicinal products for human use, Regulation (EU) 2016/679 of the European Parliament and of the Council of 27 April 2016, on the protection of natural persons with regard to the processing of personal data and on the free movement of such data, and repealing Directive 95 / 46 / EC (General Data Protection Regulation), the Spanish regulations in force on data protection, and Article 16.3 of Law 41/2002, of November 14, 2002, which regulates patient autonomy and the rights and obligations regarding clinical information and documentation.

10. Limitations of the design, data sources and methods of analysis

The primary objective has the limitation of not being a randomized study, but sets out to reflect ‘real-world’ patients, many of whom are not included in randomized trials. To minimize confounding and indication bias, we designed a propensity score matched-pair analysis. For the secondary objectives, we cannot predict the baseline MICs and mutations of the isolates, which may make it difficult to find a relationship with outcome variables if they are infrequent; nevertheless, a negative result would also be of interest.

ETHICAL CONSIDERATIONS / PROTECTION OF PARTICIPATING SUBJECTS:

1. Risk/benefit assessment for research subjects.

This study carries no risk as it is non-interventional and involves no additional or different treatment than would have been given if the patient had not participated in the study. There will be no financial benefit to patients participating in the study.

2. Considerations relating to information to participating subjects and informed consent.

A report from the Andalusian Biomedical Research Ethics Coordinating Committee is required.

This protocol complies with the principles of the Declaration of Helsinki for research projects involving human subjects.

Participants (approximately 100) selected for the microbiological (clonality, genome sequencing), biochemical and immunological analyses aimed at elucidating pathophysiological and genetic mechanisms underlying the phenotypes (Objective 5) will be informed of the nature of the study and its objectives, and will consent to participate by signing the informed consent form as a prerequisite for participation in the study.

3. Data confidentiality.

All personal data necessary for the development of the study will be collected and processed in accordance with the provisions of Regulation (EU) 2016/679 of the European Parliament and of the Council of 27 April 2016 on the protection of individuals with regard to the processing of personal data and on the free movement of such data, and repealing Directive 95/46/EC (General Data Protection Regulation), the current Spanish legislation on data protection, and Article 16. 3 of Law 41/2002, of November 14, 2002, which regulates patient autonomy and the rights and obligations regarding clinical information and documentation.

4. Interference with the prescribing habits of the physician .

This study is entirely observational and will not interfere with prescribing habits, since patients will be included in the study once their physician (independent of the research staff participating in the study) has prescribed the treatment he/she considered appropriate according to usual clinical practice.

MANAGEMENT AND REPORTING OF ADVERSE REACTIONS.

As previously indicated, this study is observational, and the type of antibiotic treatment administered will be only one of the explanatory variables that will be collected and recorded in order to achieve the proposed objectives outlined above. Therefore, IT IS NOT CONSIDERED AN OBSERVATIONAL STUDY WITH

MEDICINES, AS IT DOES NOT FALL UNDER ANY OF THE SUPPOSITIONS GIVEN IN ARTICLE 2 OF ROYAL DECREE 957/2020, OF NOVEMBER 3, REGULATING OBSERVATIONAL STUDIES ON MEDICINES FOR HUMAN USE.

PLANS FOR THE DISSEMINATION OF THE RESULTS.

The first publication will be once the inclusion period has ended and the database used by the participating centers is closed. Partial publication of results will not be allowed.

Publication of results will be in scientific journals or those available to the public, with mention of the CEIm that evaluated the study.

RESOURCES FOR THE CONDUCT OF THE STUDY AND TASK ALLOCATION; METHOD OF SUPPLY OF THE MEDICATION; FUNDING.

There is no financial compensation for patients or investigators, nor are there any undeclared sources of funding that might represent a conflict of interest for the results.

The Project has received funding from the Instituto de Salud Carlos III (Carlos III Institute of Health), with file number P121/018901 and the Fondo Europeo de Desarrollo Regional (FEDER) from the Ministerio de Hacienda y Función Pública, Gobierno de España. Has the necessary resources to carry it out.

BIBLIOGRAPHY.

1. Biedenbach DJ, et al. Occurrence and antimicrobial resistance pattern comparisons among bloodstream infection isolates from the SENTRY Antimicrobial Surveillance Program (1997 -2002). Diagn Microbiol Infect Dis 2004; 50:59 -69.
2. Fowler VG Jr, et al. Clinical identifiers of complicated *Staphylococcus aureus* bacteremia. Arch Intern Med 2003; 163:2066 -72.
3. Lesens O, et al. Role of comorbidity in mortality related to *Staphylococcus aureus* bacteremia: a prospective study using the Charlson weighted index of comorbidity. Infect Control Hosp Epidemiol 2003; 24:890 -6.
4. Lesens O, et al. Positive surveillance blood culture is a predictive factor for secondary metastatic infection in patients with *Staphylococcus aureus* bacteraemia. J Infect 2004; 48:245 -52.
5. Wyllie DH, et al. Mortality after *Staphylococcus aureus* bacteraemia in 2 hospitals in Oxfordshire. BMJ 2006; 333:281.
6. López-Cortés LE, et al. Impact of an evidence-based bundle intervention in the quality-of-care management and outcome of *Staphylococcus aureus* bacteremia. Clin Infect Dis. 2013; 57:1225-33.
7. Fowler VG, et al. Clinical identifiers of complicated Staphylococcus aureus bacteremia. Arch Intern Med. 2003. 163:2066-2072.

8. Liu et al. Clinical Practice Guidelines by the Infectious Diseases Society of America for the Treatment of Methicillin-Resistant Staphylococcus aureus Infections in Adults and Children. Clin Infect Dis. 2011;52:e18-55
9. van Hal SJ et al. Predictors of mortality in Staphylococcus aureus Bacteremia. Clin Microbiol Rev. 2012 Apr;25(2):362-86.
10. San-Juan R, et al. Pathogen-related factors affecting outcome of catheter-related bacteremia due to methicillin-susceptible *Staphylococcus aureus* in a Spanish multicenter study. Eur J Clin Microbiol Infect Dis (2017) 36:1757 -1765
11.Recker M, et al. Clonal differences in Staphylococcus aureus bacteraemia-associated mortality. NatMicrobiol.2017;2:1381-1388
12. Jenkins A, et al. Differential expression and roles of Staphylococcus aureus virulence determinants during colonization and disease. mBIO.2015; 6(1):e02272-14.
13.Giulieri SG, et al. Use of bacterial whole-genome sequencing to understand and improve the management of invasive Staphylococcus aureus infections. Expert Rev Anti Infect Ther.2016 ;14:1023-1036.
14. Fowler VG, et al. Potential Associations between Hematogenous Complications and Bacterial Genotype in *Staphylococcus aureus* Infection. The Journal of Infectious Diseases. 2007; 196-738-747.
15. Travassos, L. H., et al. Toll-like receptor 2-dependent bacterial sensing does not occur via peptido. EMBO 2004. 5: 1000 -1006.
16. Minejima E, et al. A Dysregulated Balance of Proinflammatory and Anti-Inflammatory Host Cytokine Response Early During Therapy Predicts Persistence and Mortality in Staphylococcus aureus Bacteremia. Crit Care Med. 2016; 44:671-9.
17. Pinchuk, I., et al. Staphylococcal enterotoxins. Toxins. 2010; 2:2177 -2197.
18. Wang, R., et al. Identification of cytolytic peptides as key virulence determinants for MRSA. Nat. Med. 2007. 13:1510 -1514.
19. Peschel, A., et al. Phenol-soluble modulins and staphylococcal infection. Nat. Rev. Microbiol. 2013; 11:667 -673.
19. Schreiner, J, et al. *Staphylococcus aureus* phenol-soluble modulin peptides modulate dendritic cell functions and increase in vitro priming of regulatory T cells. J. Immunol. 2013; 190:3417 -3426.
20. Stoll H, et al. Staphylococcal Enterotoxins Dose-Dependently Modulate the Generation of Myeloid-Derived Suppressor Cells. Front Cell Infect Microbiol. 2018; 8:321.
21.Uebele J, et al. *Staphylococcus aureus* Protein A Induces Human Regulatory T Cells Through Interaction With Antigen-Presenting Cells. Front Immunol. 2020; 11:581713.
22. Medina E, et al Myeloid-derived suppressor cells in infection: a general overview. J Innate Immun. 2018; 10:407 -13.
23. Gabrilovich DI, et al. Myeloid-derived suppressor cells as regulators of the immune system. Nat Rev Immunol. 2009; 9:162.
24. Pawelec G, et al. Myeloid-derived suppressor cells: not only in tumor immunity. Front Immunol. 2019; 10:1099.
25. Heim CE, et al.Myeloid-derived suppressor cells contribute to *S.aureus* orthopedic biofilm infection. J Immunol. 2014;192:3778
26. Sakaguchi S, et al. Regulatory T Cells and Immune Tolerance. Cell. 2008; 133:775 -87.
27. Golias C, et al. Review. Leukocyte and endothelial cell adhesion molecules in inflammation focusing on inflammatory heart disease. In vivo.2007;21:757-69.
28.Cowley HC, et al. Increased circulating adhesion molecule concentrations in patients with the systemic. Crit Care Med.1994.22: 29. Seymour et al, Derivation, Validation, and Treatment Implications of Clinical Phenotypes for Sepsis. JAMA. 2019; 3:21:2003-17.
30. Gutiérrez-Gutiérrez et al. Identification and Validation of Clinical Phenotypes with Prognostic Implications in Hospitalized COVID-19 Patients. A multicentre cohort-based study. Lancet Infect Dis. 2021;

PROTOCOL MODIFICATIONS.

This version is version 1.0 and has not been previously modified.

PRACTICAL CONSIDERATIONS:

1. Follow-up and interim reports.

As has been stated above, this is not an observational study with medicines for human use. Royal Decree 957/2020, of November 3, which regulates observational studies with medicines for human use is not therefore applicable. Nevertheless, the Sponsor will send the follow-up reports required by the CEIm (the Ethics Committee for Research with medicines) for approval.

2. Dissemination of the results

The results of this study will be communicated at different international, national and regional conferences specialized in Infectious Diseases and/or clinical microbiology.

They will also be submitted for publication in international journals with impact factor.

ANNEXES (not included in the protocol):

Annex 1: Data collection sheet.

Annex 2: Commitment of the coordinating investigator.

Annex 3: Agreement / Decision of the 1° CEI (Ethics Committee for Research) that evaluated the study.

Annex 4: Subject information sheet (only for patients selected for objective 5).

Annex 6: Informed consent form (only for patients selected for objective 5).

Annex 7: Financial report.
