# Supplementary material for: Identification and validation of clinical phenotypes in Staphylococcus aureus bloodstream infection and their association with mortality (FEN-AUREUS study)
Source: eClinicalMedicine. 2025 May 7;83:103240. doi: 10.1016/j.eclinm.2025.103240 (PMC12235390; doi:10.1016/j.eclinm.2025.103240)
Supplement: Supplementary ISAC Study Group author list [file mmc2.docx]

**Supplementary Appendix – list of investigators from the ISAC Study Group**

Marina de Cueto, Isabel Morales, Jesús Rodríguez-Baño (Hospital Universitario Virgen Macarena, Sevilla, Spain), Eu Suk Kim, Chung-Jong Kim, Chang Kyung Kang, Jung In Park (Seoul National University Bundang Hospital, South Korea), Christian Bernasch, Danuta Stefanik, Norma Jung, Martin Hellmich (University of Cologne, Cologne, Germany), Peter Wilson, Anna Reyes, Saadia Rahman, Victoria Dean (University College London Hospitals NHS Foundation Trust, London, UK), Miguel Marcos and Hugo Guillermo Ternavasio-de la Vega (University Hospital of Salamanca-USAL-IBSAL, Salamanca, Spain), Estée Török, Theodore Gouliouris, Luke Bedford (University of Cambridge, Cambridge, UK), José L. Pérez, Enrique Ruiz de Gopegui, Maria Luisa Martín-Pena (Hospital Universitario Son Espases, Palma de Mallorca, Spain), Susan Hopkins (Royal Free London NHS Foundation Trust, London, UK), Karuna Lamarca, Beatriz Mirelis, Mercedes Gurgui Ferrer (Hospital de la Santa Creu i Santa Pau, Barcelona, Spain), Vance G. Fowler, Felicia Ruffin (Duke University Hospital, Durham, USA), José Miguel Cisneros Herreros, José A. Lepe, Cristina Roca (Hospital Universitario Virgen del Rocío, Sevilla, Spain), James R Price, Angela Dunne, Laura Behar (Brighton and Sussex University Hospitals NHS Trust.
