# Supplementary material for: Identification and validation of clinical phenotypes in Staphylococcus aureus bloodstream infection and their association with mortality (FEN-AUREUS study)
Source: eClinicalMedicine. 2025 May 7;83:103240. doi: 10.1016/j.eclinm.2025.103240 (PMC12235390; doi:10.1016/j.eclinm.2025.103240)
Supplement: Supplementary INSTINCT study group author list [file mmc3.docx]

**Supplementary Appendix – list of investigators from the Invasive STaphylococcus aureus INfection CohorT (INSTINCT) Study Group**

Achim J. Kaasch, Harald Seifert, Hanna Birkholz, Katharina Achilles, Andreas Langhorst, Stephan Neumann, Georg Peppinghaus (Uniklinik Köln); Siegbert Rieg, Winfried V. Kern, Marc-Fabian Kupper, Gabriele Peyerl-Hoffmann, Christian Theilacker (Universitatsklinikum Freiburg).
