# Supplementary material for: Identification and validation of clinical phenotypes in Staphylococcus aureus bloodstream infection and their association with mortality (FEN-AUREUS study)
Source: eClinicalMedicine. 2025 May 7;83:103240. doi: 10.1016/j.eclinm.2025.103240 (PMC12235390; doi:10.1016/j.eclinm.2025.103240)
Supplement: Supplementary FEN-AUREUS study group author list [file mmc4.docx]

**Supplementary Appendix – list of investigators from the CIBERINFEC and FEN-AUREUS study group**

Elena Ruíz-Gutiérrez and Lola Cubero-Aranda (Hospital Universitario Virgen de Macarena/Instituto de Biomedicina de Sevilla (IBiS)); R. Herrero and Ana I. Aller (Hospital Universitario de Valme/Instituto de Biomedicina de Sevilla (IBiS)); Pilar González de-la-Aleja and Livia Giner-Olcina (Infectious Diseases Unit, Dr. Balmis General University Hospital, Alicante Institute for Health and Biomedical Research (ISABIAL), Alicante, Spain); Andrés Martín-Aspas and Fátima Galán (Unidad de Enfermedades Infecciosas del Hospital Universitario Puerta del Mar, Cádiz); Marina Murillo-Pineda (Instituto de Investigación e Innovación Biomédica de Cádiz) and Rubén Lobato-Cano (Unidad de Enfermedades Infecciosas y Microbiología Clínica, Hospital Universitario de Jerez); Giacomo Casalini and Maria Vittoria Cossu (Department of Infectious Diseases, ASST FBF Sacco, Milán, Italy); José Miguel Cisneros and Adrián Pulido (Clinical Unit of Infectious Diseases, Microbiology and Parasitology/ Instituto de Biomedicina (IBIS), Virgen del Rocío University Hospital, Seville, Spain).
